# MIF inhibitor ISO-1 attenuates ischemia-reperfusion injury in a rat model of donation after circulatory death liver transplantation

**DOI:** 10.1371/journal.pone.0361214

**Published:** 2026-10-26

**Authors:** Deniz Uluk, Wenjia Liu, Decan Jiang, Christian Stoppe, Franziska Alexandra Meister, Sandra Kraemer, Christian Beckers, Alexander Theissen, Christian Bleilevens, Jan Bednarsch, Ulf Peter Neumann, Rene Hany Tolba, Zoltan Czigany

**Affiliations:** 1 Department of General Surgery, Division of Transplantation, Medical University of Vienna, Vienna, Austria; 2 Institute for Laboratory Animal Science and Experimental Surgery, Faculty of Medicine, University Hospital RWTH Aachen, Aachen, Germany; 3 Department of Anaesthesiology, Intensive Care, Emergency and Pain Medicine, University Hospital, Würzburg, Germany; 4 Department of Cardiac Anaesthesiology and Intensive Care Medicine, Deutsches Herzzentrum der Charité, Berlin, Germany; 5 Charité-Universitätsmedizin Berlin, Corporate Member of Freie Universität Berlin, Humboldt-Universität zu Berlin, Berlin, Germany; 6 Department of Surgery and Transplantation, University Hospital RWTH Aachen, Aachen, Germany; 7 Department of Thoracic and Cardiovascular Surgery, University Hospital RWTH Aachen, Aachen, Germany; 8 Cardiovascular Critical Care & Anesthesia research and evaluation (3CARE), University Hospital RWTH Aachen, Aachen, Germany; 9 Department of Intensive Care Medicine, RWTH Aachen University, Aachen, Germany; 10 Department of General, Visceral, Vascular, and Transplantation Surgery, University Hospital Essen, Essen, Germany; 11 Faculty of Medicine, University Duisburg-Essen, Essen, Germany; Anhui Polytechnic University, CHINA

## Abstract

Macrophage migration inhibitory factor (MIF) is an early mediator of ischemia–reperfusion injury (IRI), however, the evidence on its role in orthotopic liver transplantation (OLT), particularly with donation after circulatory death (DCD) grafts is limited. Given its key regulatory function, this study investigated the effects of IRI on graft injury and assessed whether the use of ISO-1, a small-molecule MIF-inhibitor, affect IRI in a rat DCD-OLT model. 196 male Lewis rats were randomized into DBD control, DCD-30 min, DCD-60 min, DCD-30 + ISO-1, and DCD-30 + vehicle groups (n = 7/group/time-point). ISO-1 was administered before controlled cardiac arrest (0–60 min) and 3 h cold preservation before arterialized OLT. Animals were sacrificed at 1, 3, 24, and 168 h post-reperfusion. DCD grafts showed marked injury, with the DCD-60 group exhibiting lethal IRI. ISO-1 significantly reduced early hepatocellular injury compared with vehicle (AST:294 ± 63 vs 867 ± 211 IU/L, p = 0.017) and resulted in values comparable to DBD controls. Serum HMGB-1 (11 ± 2.8 vs 26 ± 6.2 pg/mL, p = 0.045) and MIF (45 ± 5.3 vs 144 ± 27.5 ng/mL, p = 0.034) levels were similarly decreased following ISO-1 application. ISO-1 improved graft microcirculation at 24 h, attenuated histological injury, and was associated with an altered time course of CD163 + macrophage infiltration. ISO-1 pre-treatment attenuated hepatocellular injury and was associated with improved graft microcirculation and reduced DAMP release, supporting further mechanistic and translational evaluation in DCD liver transplantation.

## Introduction

Liver transplantation using allografts from donation after circulatory death (DCD) has led to the life-saving and substantial expansion of the donor pool over the past decades [1]. While offering the potential to increase the number of livers offered for transplantation, ischemia-reperfusion injury (IRI) during DCD organ donation still represents a major risk factor for inferior post-transplant outcomes [2]. Even though, state of the art organ preservation technology with the use of normothermic regional perfusion and machine perfusion has largely improved the overall donor pool utilization [3,4], allografts from DCD donors besides their short-term risks (such as primary non-function, post-reperfusion syndrome, early allograft dysfunction, graft-related complication), are also often associated with inferior long-term outcomes, due to biliary complications, acute- and chronic rejection, and graft loss [5].

First described in 1966, macrophage migration inhibitory factor (MIF) is a pro-inflammatory cytokine that functions by inhibiting the random migration of macrophages, thereby retaining them at sites of injury and inflammation [6,7]. MIF is a pleiotropic cytokine which is rapidly released upon stimulation from intra-plasmatic vesicular pools of various cell-types, including immune cells, hepatocytes, endothelial cells [8]. After activation and secretion, MIF has the ability to promote tissue injury and initiate inflammatory responses on the humoral and cellular level [8]. The Vienna group has shown for the first time in the clinical setting that human liver grafts release MIF and higher concentrations of MIF are associated with allograft dysfunction and acute kidney injury after OLT [8–10]. Although, our group and others have demonstrated to role of MIF in IRI in various organs [8–14], clinical and experimental scenarios, to the best of our knowledge, no data are available on the modulation of MIF with its inhibitor ISO-1 in the context of IRI and OLT.

Therefore, we hypothesized that donor pre-treatment with the MIF inhibitor ISO-1 might attenuate ischemia-reperfusion injury, improve graft function, and modulate the early inflammatory response in a rat model of DCD liver transplantation.

## Methods

### Animals and study design

This study was designed, conducted and reported based on the ARRIVE (Animal Research: Reporting of In Vivo Experiments) and based on our review article published on reporting standard in experimental studies in rat liver transplantation [15]. The experiments were approved by Governmental Animal Care and Use Committee (LANUV NRW “Landesamt fuer Natur, Umwelt und Verbraucherschutz Nordrhein-Westfalen”, Recklinghausen, Germany, ID: 81–02.04.2017. A 414). All rats in the study received the best possible care according to the principles of the “Guide for the Care and Use of Laboratory Animals” (8th edition, NIH Publication, 2011, USA). Experiments were performed in accordance with the institutional guidelines and the German federal law regarding the protection of animals and the EU Directive 2010/63. Every staff member involved has received all required training according to the local requirements and standards of the Society for Laboratory Animal Science (GV-SOLAS) and Federation of European Laboratory Animal Science Associations (FELASA).

All rats were housed in the animal facilities and kept under specific pathogen-free conditions at least 7 days before experiments according to the guidelines of the “Federation for Laboratory Animal Science Associations” (FELASA; www.felasa.eu) in a temperature- and humidity-controlled environment with a 12/12 day/night cycle. Standard food for laboratory rats (Sniff GmbH, Soest, Germany) and water were granted ad libitum. Confounders were controlled in a way that all personnel involved in the daily care of the animals was blinded for the experimental groups throughout the experiments.

Two experiments were conducted in this study: 196 male Lewis rats (LEW/OrlRj; Janvier Labs, Le Genest Saint Isle, France) were divided into 5 groups based on an *a priori* sample size calculation (alpha = 0.05; 1-beta = 0.8, peak 73% ALT difference between groups based on previous studies of our group) and used to perform 98 arterialized whole-graft isogenic OLTs, (n = 7/group/time point). To comply with the 3R principles, rats in group 1 were also designed as the control group for experiment II to reduce the number of animals used (Reduction principle – 3R). Fig 1 depicts the study protocol.

**Fig 1:**
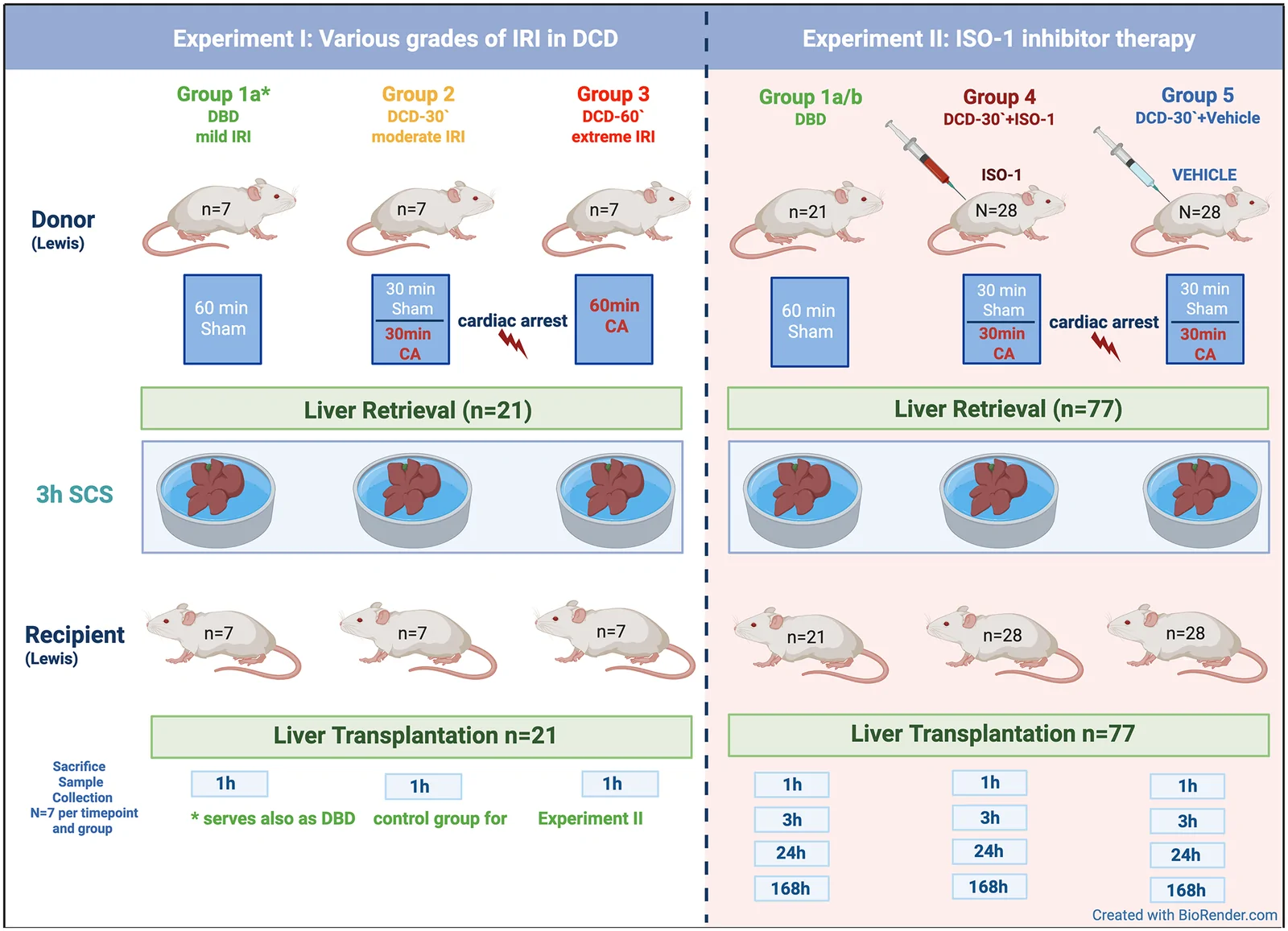
Study flowchart. Animals were randomized into five experimental groups (DBD control group, DCD-30’ group, DCD-60’ group, ISO-1 group, Vehicle group). 60 minutes sham period, 30 minutes sham period + 30 minutes WIT, and 60 minutes WIT were performed in group 1, 2, 4, 5, and 3 before liver retrieval, respectively. Additionally, in group 4 and 5, ISO-1, and DMSO 5% were injected 30 min before laparotomy, respectively. Following liver retrieval, grafts were stored for 3 hours in HTK solution at 4-degree for cold preservation and implanted into the recipient animals. Animals were sacrificed after 1, 3, 24, and 168 hours of reperfusion for sample collection and analysis. Animals in group 1a were also included into the control group for experiment II to reduce the number of animals used. IRI: ischemia reperfusion injury; SCS: static cold storage; HTK: histidine tryptophane ketoglutarate solution; DBD: donation after brain death; DCD: donation after circulatory death; DMSO: dimethylsulfoxide; OLT: orthoptic liver transplantation; WIT: warm ischemia time.

#### Experiment I.

- Group-1a (DBD control group), before liver retrieval, 60 minutes sham period of anesthesia was performed without further intervention, followed with OLT.
- Group-2 (DCD-30’ group), 30 minutes sham period together with 30 minutes of cardiac arrest was performed before liver retrieval, followed with OLT. Cardiac arrest was achieved in all DCD groups via phrenotomy.
- Group-3 (DCD-60’ group), 60 minutes of cardiac arrest was performed before liver retrieval, followed by OLT.

Following 1 hour of “on-table” reperfusion. Animals were sacrificed in deep anesthesia by exsanguination via the vena cava following the assessment of graft perfusion. Sample were collected for further analysis. Due high mortality after 60 min of cardiac arrest and DCD liver transplantation, no long-term survival groups have been established in Experiment I.

#### Experiment II.

- Group-1a + b (DBD control group), similarly, to Experiment I., donor animals of this group underwent 60 minutes of sham period and no further treatment. Recipient animals were sacrificed after 1/3/24/168-hours (n = 7/group/time-point) of graft reperfusion and acted as control group. Data from the 1-hour reperfusion group was used from Experiment I.
- Group-4 (ISO-1 group, DCD-30’ + ISO-1), ISO-1 (Calbiochem, CAS 478336-92-4, San Diego, CA) (20 mg ISO-1 /KG kg) was applied intraperitoneally to the donor animal directly after anesthesia induction (1 hour before cardiac arrest). ISO-1 was diluted with minimal amounts of dimethylsulfoxide (DMSO) (23 mg/mL solution), which was then further diluted with 0.9% NaCl to a final DMSO concentration of 5.2%. Thirty minutes sham period combined with 30 minutes cardiac arrest was performed before liver retrieval, followed by OLT.
- Group-5 (Vehicle group, DCD-30’ + Vehicle) DMSO 5% (DMSO diluted by 0.9% NaCl to a final DMSO concentration of 5.2%) intraperitoneally to the donor animal directly after anesthesia induction (1 hour before cardiac arrest). Thirty minutes sham period combined with 30 minutes cardiac arrest was performed before liver retrieval, followed by OLT.

Following the corresponding observation period (1/3/24 or 168 hours) the animals were put into anesthesia and a relaparotomy was carried out to assess graft circulation and collect samples. Animals are sacrificed in deep anesthesia by exsanguination via the vena cava.

### Liver transplantation

All surgeries were performed by the same experienced microsurgeon (Z.C.) and at the same time of day to avoid disturbing effects of circadian rhythm. All rats were anesthetized with 2vol% isoflurane (Forane; Abbott GmbH, Wiesbaden, Germany) and buprenorphine (0.03 mg/kg) (Temgesic; EssexPharma, Haar, Germany) during all the surgical interventions and samples collection. Surgical details of arterialized OLT in rats used in the present study were extensively described by our group earlier [16].

Briefly, in all donor animals, the liver was perfused via the portal vein with 60 mL of 4˚C Histidine-tryptophan-ketoglutarate (HTK) solution (Custodiol; Dr Franz Koehler Chemie GmbH, Bensheim, Germany) at a hydrostatic pressure of 20 cm H_2_O. Liver was then retrieved *en bloc* and preserved in HTK solution at 4°C for 3 hours cold preservation and meanwhile, back-table procedures were performed.

During recipient surgery, following hepatectomy reconstruction, of the suprahepatic inferior vena cava and portal vein were carried out by end-to-end anastomosis using continuous sutures (Prolene 7–0; Ethicon, Somerville, NJ, USA) and 3.5 mm long 14-gauge polyethylene cuff with circumferential grooves (Vasofix 14G; B. Braun), respectively. Graft was perfused immediately after the portal vein was reconstructed. Infrahepatic inferior vena cava was then reconstructed in the same method as using continuous sutures (Prolene 8–0; Ethicon). For graft re-arterialization and bile duct reconstruction, 24-gauge polyethylene stents (Vasofix 24G; B. Braun) were used in a length of 3.5 mm and 5 mm, respectively. In general, the hepatectomy took around 30 minutes, and the anhepatic time was within 20 minutes. At the end of the surgery, the incision was closed in two layers using continuous 4−0 sutures (Vicryl 4−0; Ethicon). Intraoperative fluid administration was described in detail by our team elsewhere [17].

### Post-operation managements

Recipient rats recovered from anesthesia and surgery in a special intensive care unit cage (Vetario; Brinsea Products Ltd, North Somerset, UK) with warmed air (30°C – 35°C) and oxygen supplementation. Antibiotics and analgesia were administered by subcutaneous injection of cefuroxime sodium (16 mg/kg) (Cefuroxim Fresenius; Fresenius Kabi Deutschland GmbH, Bad Homburg, Germany) and buprenorphine (0.03 mg/kg) (Temgesic; EssexPharma, Haar, Germany) every 24 hours until POD 3. Postoperative follow-up and humane endpoint scoring have been performed twice daily as described elsewhere by our working group [16]. The specific criteria used to determine when animals should be euthanized has been attached as online supplement (S2_Score_Sheet).

### Serum transaminases and graft circulation

Blood samples from the inferior vena cava, were centrifuged (4°C, 10 minutes, 2,500 rpm) and then levels of alanine aminotransferase (ALT), aspartate aminotransferase (AST), lactate dehydrogenase (LDH) were measured using standard photometric procedures in an automated analyzer (Vitros 250; Johnson and Johnson, Neuss, Germany). Serum transaminases have been expressed as IU/L.

Graft microcirculation and portal venous flow were measured using an O2C device (O2C-oxygen to see device, LF1 surface probe; LEA Medizintechnik GmbH, Giessen, Germany) and transit-time perivascular flowmeter (T403 device, MA2PSB flow probe; Transonic Systems, Inc., Ithaca, NY, USA), respectively. Details of measurement have been described elsewhere by our working group [16].

### Histology and immunohistochemistry

Liver samples were harvested from mediate lobe for histopathology. First, the excised tissue was fixed in 10% neutral buffered formalin. Second, 4 mm to 6 mm thick Slides embedded by paraffin were stained with H&E (hematoxylin and eosin). All slides were evaluated using a semiquantitative scoring system adopted from Suzuki et al. by an experienced veterinary pathologist in a blinded fashion, assessing congestion, vacuolization, and hepatocyte necrosis [16]. To simplify the results, a total score was generated (none = 0; minimal = 1; mild –30% = 2; moderate 30–60% = 3; severe >60% = 4). Immunohistochemical double-staining of FFPE liver tissue sections was carried out using rat recombinant anti-CD163 monoclonal antibodies (Abcam, Cambridge, UK, EPR19518, ab289979).

Ten randomly chosen, nonoverlapping fields (400x magnification) were scored using light microscopy (Leica DM 2500; Leica Microsystems GmbH, Wetzlar, Germany).

### mRNA expression by reverse-transcription polymerase chain reaction (RT-PCR)

To measure the messenger ribonucleic-acid (mRNA) expression in the liver tissue, RT-PCR was performed using TaqMan technology. Specific TaqMan probes (Thermo Fisher Scientific, Waltham, MA, USA, 4331182, Rn00821234_g1) were used on a StepOne Plus Cycler (Applied Biosystems). Methods in detail were described elsewhere by our study group [18].

### Protein expression by western blot (WB)

Protein expression in liver tissue was detected using WB technique (DC-Protein Assay Kit, BIO-RAD Laboratories, Munich, Germany) as described previously by our group [16]. Incubation with specific antibodies against MIF (Cell Signaling Technology, Danvers MA, USA) was performed. The results were displayed as integrated density value (IDV), relative to GAPDH (Glyceraldehyde 3-phosphate dehydrogenase). Western blot images have been grouped only for better visualization, raw data have been provided as online supplement (Supplementary Data 1)

### HMGB-1 and MIF enzyme-linked immunosorbent assay (ELISA)

Serum HMGB-1 and MIF levels were assessed using commercially available rat HMGB-1 and MIF ELISA kits (ST51011, IBL Int GmbH, Hamburg, Germany; CSB-E07293r, Cusabio Technology LLC, Houston,Tex, USA) according to the manufacturer’s instructions.

### Statistical analysis

Two-way analysis of variance (ANOVA) and Bonferroni post hoc correction were performed to analyze changes in time-dependent parameters and between-group differences in each timepoint. Non-parametric Kruskal–Wallis H test was used to test the differences between three groups.

A *p*-value < 0.05 was considered as significance, and adjusted P-value was applied when data have been corrected with multiple comparisons. All statistical analyses were calculated and plotted by GraphPad Prism 10 (GraphPad Software Inc, San Diego, Calif., USA).

## Results

General characteristics of experimental animals and surgical quality indicators, anhepatic time (p = 0.08 between groups, Kruskal–Wallis H test), total surgery time (p = 0.08), cold ischemia time (p = 0.12) did not differ significantly between control and experimental groups as illustrated in Table 1.

**Table 1:** General characteristics of the experimental animals and intra-operative parameters for quality assurance.

	DBD	DCD-30’	DCD-60’	DCD-30’ + ISO-1	DCD-30’ + vehicle	P Value
Donor BW (g)	277.1 ± 3.5	285.8 ± 4.1	274.2 ± 6.3	267.4 ± 5.0	263.3 ± 4.0	0.08
Recipient BW (g)	283.6 ± 2.8	281.3 ± 6.1	274.8 ± 6.5	270.9 ± 4.2	269.4 ± 2.8	0.06
Graft weight (g)	11.32 ± 0.2	12.59 ± 0.3	11.63 ± 0.4	11.53 ± 0.2	11.92 ± 0.2	0.07
CIT (min)	178.9 ± 2.9	185.0 ± 1.5	180.5 ± 2.4	185.3 ± 1.0	177.3 ± 1.5	0.12
Anhepatic time (min)	16.3 ± 0.3	16.6 ± 0.3	17.1 ± 0.6	15.5 ± 0.3	15.8 ± 0.4	0.08
Total surgery (min)	80.3 ± 3.4	93.3 ± 1.4	94.6 ± 2.9	80.1 ± 3.2	82.3 ± 4.2	0.08

Values are expressed as mean ± standard error of the mean. Abbreviation used: BW-body weight, CIT-Cold ischemia time.

### Experiment I – Graft injury in DCD liver transplantation using livers subjected to various degrees of IRI

To characterize different degrees of graft injury in our model following DBD, DCD with 30- or 60-minutes cardiac arrest, we monitored serum AST, ALT, and LDH levels and assessed graft macro- and microcirculation at sacrifice in each recipient (Fig 2).

**Fig 2:**
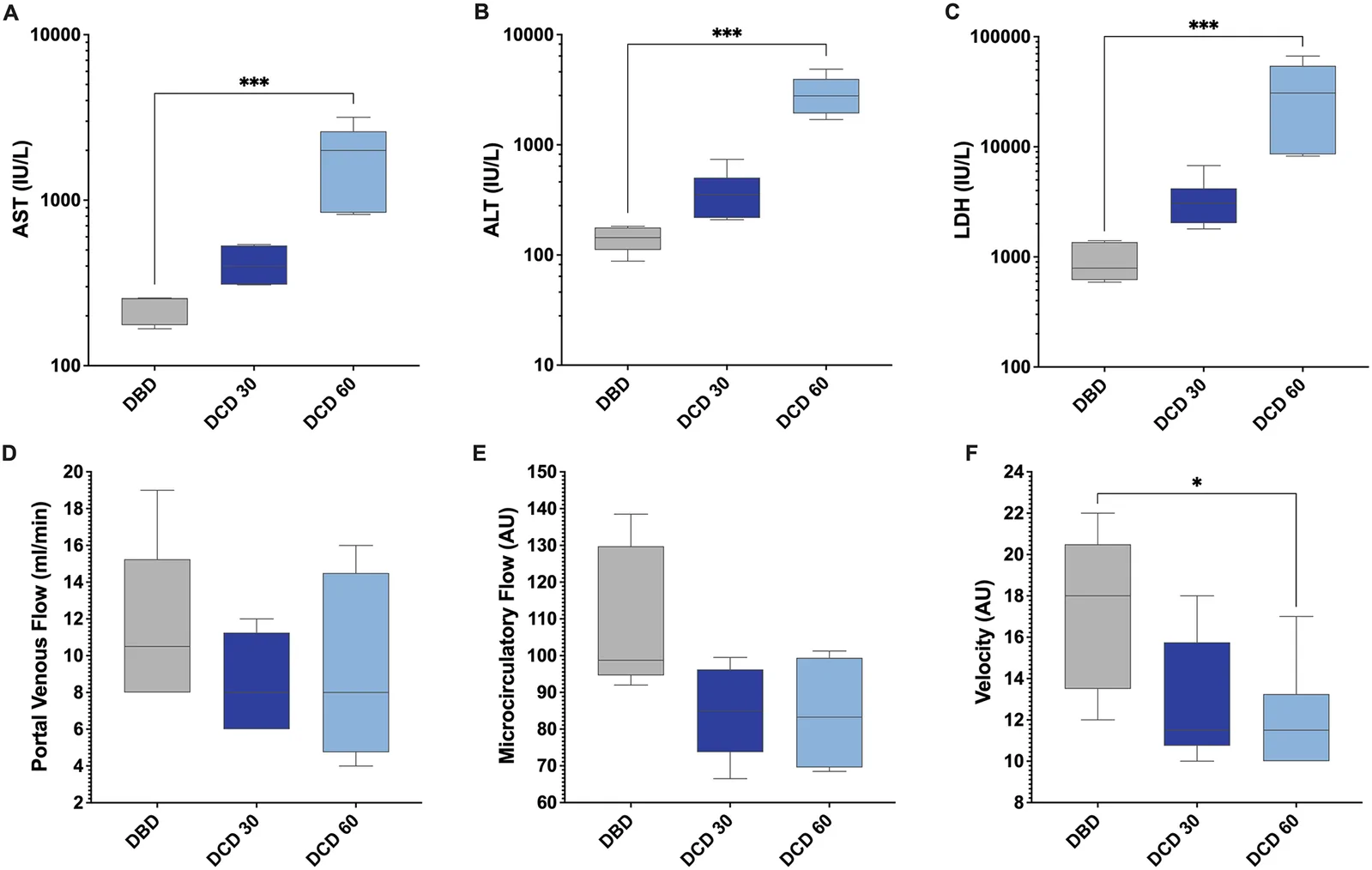
Serum markers of hepatocellular injury (A-C) and graft macro- and microcirculation (D-F) following OLT subjected to various degrees of IRI. *p < 0.05, ***p < 0.001; values are expressed as median & interquartile range; n = 7/group/time point. Abbreviations used: AST-Aspartate aminotransferase; ALT-Alanine aminotransferase; LDH-Lactate dehydrogenase; AU-Artificial unit.

While animals of the DBD group only showed a mild increase in serum transaminases (AST: 224 ± 20 IU/L / ALT: 142 ± 15 IU/L; Fig 2) and LDH (LDH: 924 ± 359 IU/L; Fig 2) following reperfusion, DCD groups were characterized by a moderate and extreme hepatocellular injury following 30- and 60-minutes of cardiac arrest, respectively (Fig 2). Meanwhile, a statistically strongly significant difference was found between the DCD 60 and DBD groups (AST: DCD 60 vs DBD, 1876 ± 373 vs 224 ± 20 IU/L, p = 0.0005 // ALT: DCD 60 vs DBD, 2912 ± 542 vs. 142 ± 15 IU/L, p = 0.0005 // LDH: DCD 60 vs DBD, 32619 ± 10076 vs 924 ± 147 IU/L, p = 0.0003, Fig 2), the DCD 30 and DCD 60 groups only demonstrated a clear tendency of between group differences (p = 0.1188 // p = 0.2162 // p = 0.1547, respectively, see Fig 2).

The macro- and microcirculation of the DCD grafts was inferior compared to the DBD groups, especially microcirculatory flow and velocity showed poor results in both DCD groups (Velocity: DCD 60 vs DBD, 12 ± 1.1 vs 17 ± 1.5 AU, p = 0.0385, Fig 2).

### Experiment II – ISO-1 MIF inhibitor attenuates biochemical injury and DAMPs release in DCD liver transplantation

Using the model of moderate IRI with 30 minutes cardiac arrest for the pharmacological treatment with ISO-1, acute hepatocellular damage after 1 hour of reperfusion, observed in the DCD Vehicle group was markedly reduced in the ISO-1 group (AST: DCD ISO-1_1hour_ vs DCD Vehicle_1hour_, 294 ± 63 vs 867 ± 211 IU/L, p = 0.017 // ALT: DCD ISO-1_1hour_ vs DCD Vehicle_1hour_, 246 ± 157 vs 572 ± 514 IU/L, p = 0.075 // LDH: DCD ISO-1_1hour_ vs DCD Vehicle_1hour_, 2231 ± 1529 vs 4365 ± 1741 IU/L, p = 0.003, Fig 3). In particular, AST, ALT and LDH levels of the ISO-1 treated animals were more comparable with those of the DBD group rather than with the values of the DCD Vehicle group with no significant difference between DBD and DCD ISO-1 (ALT: p = 0.980; AST: p = 0.081; LDH: p = 0.230, Fig 3), suggesting the potent effects of ISO-1 in this model (Fig 3).

**Fig 3:**
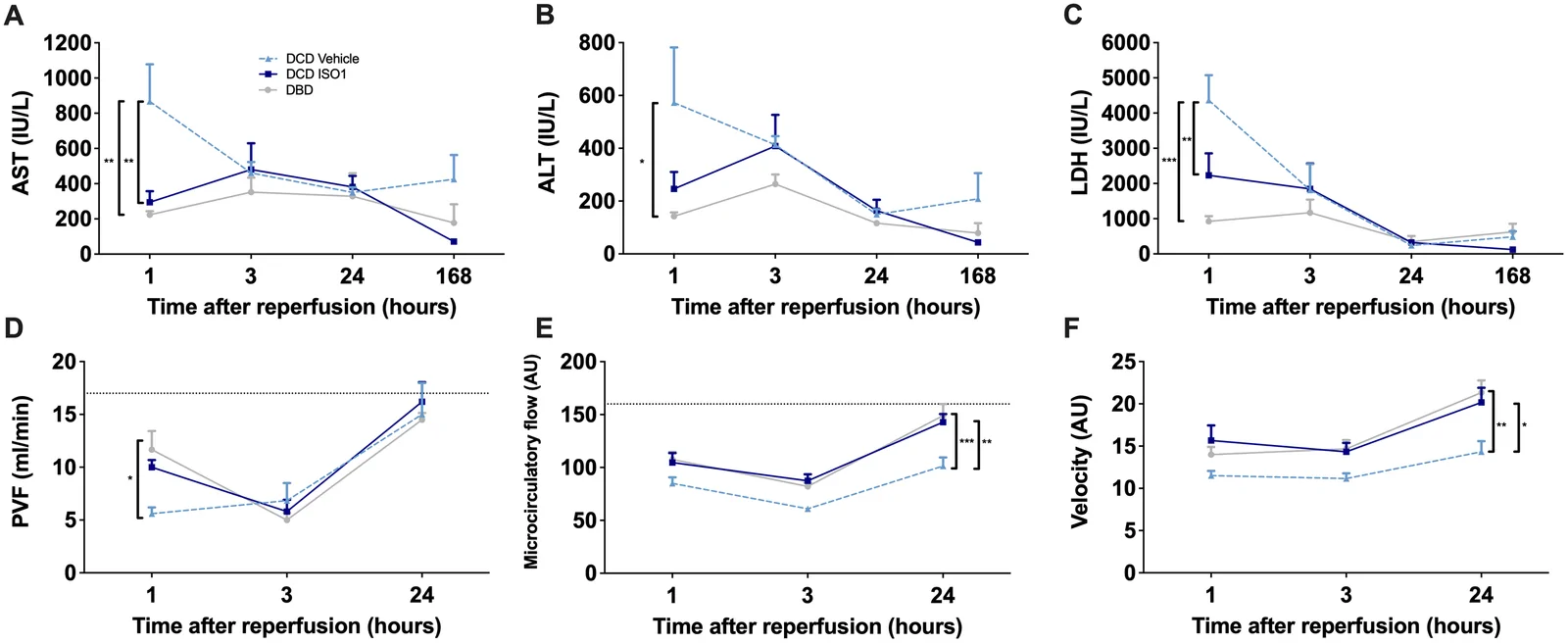
Serum markers of hepatocellular injury (A-C) and graft macro- and microcirculation (D-F) following the treatment with ISO-1 versus Vehicle. *p < 0.05, **p < 0.01, ***p < 0.001; values are expressed as mean ± standard error of the mean; n = 7/group/time point. Abbreviations used: AST-Aspartate aminotransferase; ALT-Alanine aminotransferase; LDH-Lactate dehydrogenase; AU-Artificial unit; PVF-Portal venous flow.

Serum levels of the key DAMP HMHB-1 protein were likewise markedly increased in the DCD Vehicle group, while the DCD ISO-1 and DBD groups were characterized by largely similar curves and significantly lower values (HMGB-1: DCD ISO-1_1hour_ vs DCD Vehicle_1hour_, 11 ± 2.8 vs 26 ± 6.2 pg/mL, p = 0.045, Fig 4).

**Fig 4:**
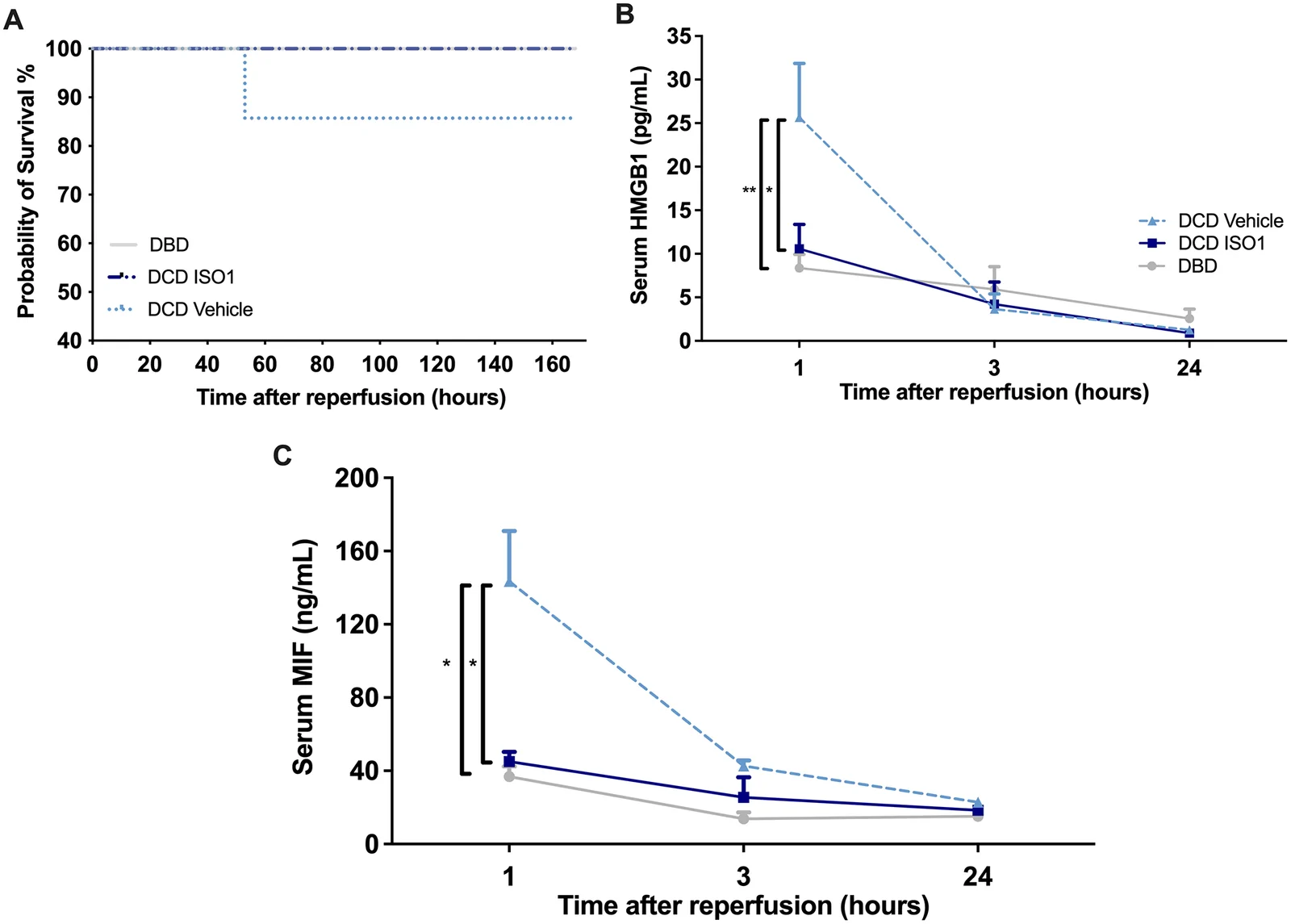
Recipient survival (A), serum HMGB-1 (B) and MIF levels (C). One animal in the DCD Vehicle group has died due to biliary complications and cholangitis. *p < 0.05, **p < 0.01, ***p < 0.001; values are expressed as mean  ±  standard error of the mean; n = 7/group/time point. Abbreviations used: HMGB1-High mobility group box 1 protein.

### ISO-1 MIF inhibitor improves graft macro- and microcirculation

To assess the effects of IRI and ISO-1 on graft circulation, portal venous flow and liver microcirculation were measured (Fig 3). While portal venous flow was higher in DBD and DCD ISO-1 groups in the very acute phase (PVF: DCD Vehicle_1hour_ vs DBD_1hour_, 5.6 ± 1.3 vs 12 ± 4.3 ml/min, p = 0.039, Fig 3), significant differences in microcirculation flow and velocity were more prominent in a later phase, after 24 hours of reperfusion (O2C Flow: DCD ISO-1_24hours_ vs DCD Vehicle_24hours_, 142 ± 17 vs 101 ± 18 AU, p = 0.002 // O2C Velocity: DCD ISO-1_24hours_ vs DCD Vehicle_24hours_, 20 ± 4 vs 14 ± 3 AU, p = 0.014, Fig 3). At this time point both the DBD and DCD ISO-1 groups have showed a significantly improved graft microcirculation compared to the DCD Vehicle group (Fig 3).

### Survival

Registration of the clinical status of the animals was performed every 12 h using a humane endpoint score sheet based on Kanzler et al. as described previously by our group [19]. In each group 7 animals underwent liver transplantation to assess long-term survival over one week. Except one animal which developed severe biliary complication and cholangitis and was sacrificed without delay as humane endpoint was reached (S2 Table) and to collect samples, all other animals have survived the designated observation period. Accordingly, no animal has died before meeting criteria for euthanasia. No significant difference in one week survival was registered (log-rank p = 0.368, Fig 4).

### ISO-1 MIF inhibitor mitigates histopathological injury and impacts macrophage polarization

Morphological damage was analyzed after 24 hours of reperfusion, as based on previous studies of our group, this time point represents the peak of histological injury. Mild and moderate IRI with tissue degeneration, inflammation, vacuolization and necrosis was found on all tissue slied in the experimental groups. However, the DCD Vehicle group showed a more extensive damage with a higher total score compared to DBD (Suzuki total score median and IQR: DCD Vehicle_24hours_ vs DBD_24hours,_ 5.8 and 6.6–5.5 vs 3.8 and 5.3–3.3, p = 0.022, Fig 5). The DCD ISO-1 animals have demonstrated a histological injury which did not differ significantly from that of the DBD group (Suzuki total score: DCD ISO-1_24hours_ vs DBD_24hours_, 5.8 and 6.6–5.5 vs 3.8 and 5.3–3.3, p = n.s., Fig 5).

**Fig 5:**
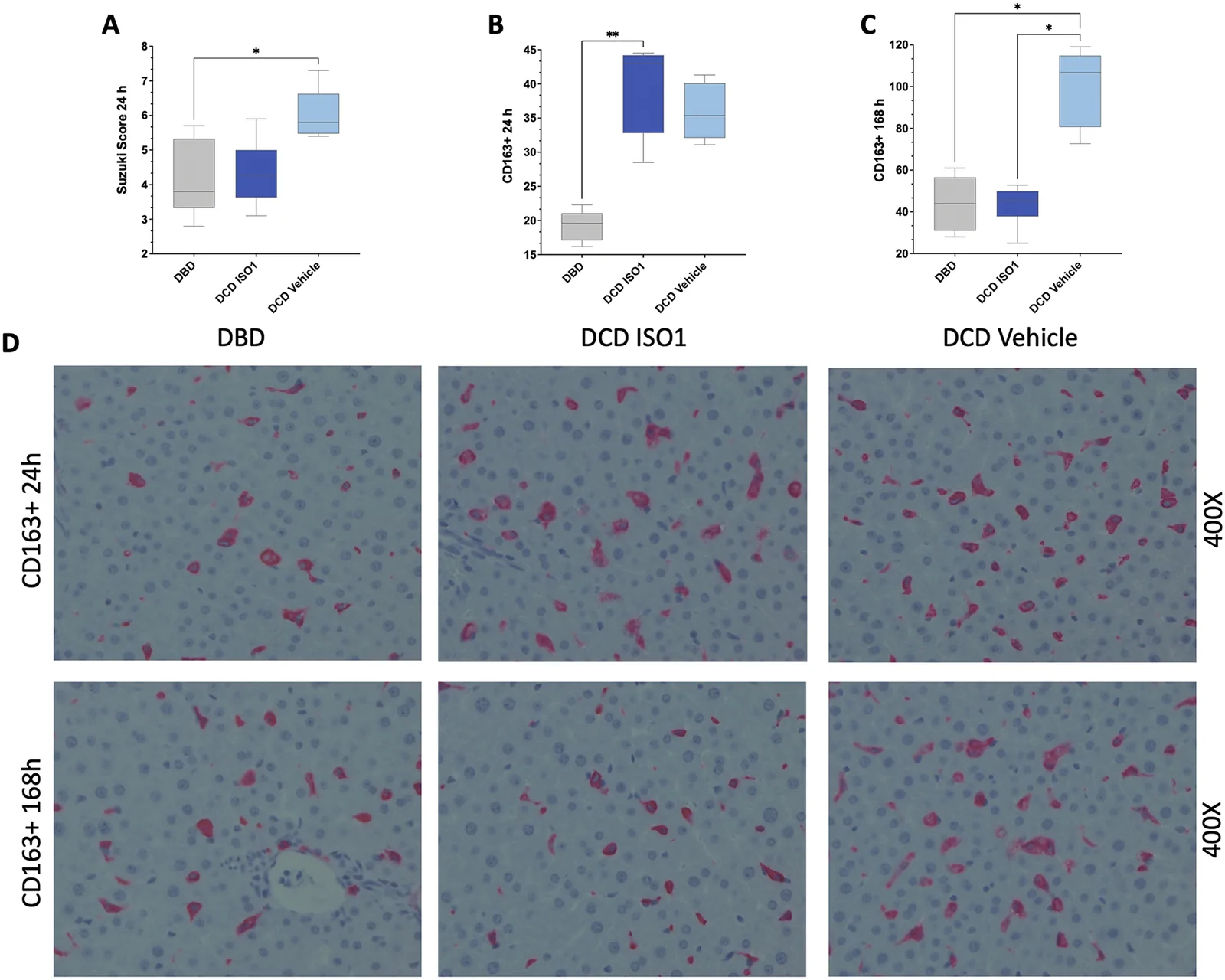
Histopathology and CD163 immunohistochemistry. Histopathological injury was assessed on HE stained slides and light microscopy using the modified Suzuki score. The number of activated M2 macrophages was evaluated using CD163 immunohistochemistry (B-D). *p < 0.05, **p < 0.01, ***p < 0.001; values are expressed as mean ± standard error of the mean; n = 7/group/time point.

The number of activated M2 type macrophages in the liver was increased in both DCD ISO-1 and Vehicle groups after 24 hours of reperfusion, however, a significant difference could only be registered between the DCD ISO-1 and DBD groups (CD163 + 24 hours: DCD ISO-1_24hours_ vs DBD_24hours_, 40 ± 4 vs 19 ± 2 cells/10 HPF, p = 0.003, Fig 5). After the one-week observation period the number of CD163 + cells were persistently increased in the DCD Vehicle group, while it was reduced to the level of the DBD animals in the DCD ISO-1 group (CD163 + 168 hours: DCD ISO-1_168hours_ vs DCD Vehicle_168hours_, 43 ± 13 vs 100 ± 19 cells/10 HPF, p = 0.016, Fig 5).

### MIF mRNA expression, serum and tissue protein levels

Despite some trends toward higher values in the vehicle group, tissue encoding mRNA expression of MIF was not different between the experimental groups over the 1/3/24-hour observation period (e.g., MIF RNA: DCD ISO-1_1hour_ vs DCD Vehicle_1hour_, 1.1 ± 0.1 vs 1.5 ± 0.2 RQ, p = 0.392, Fig 6).

**Fig 6:**
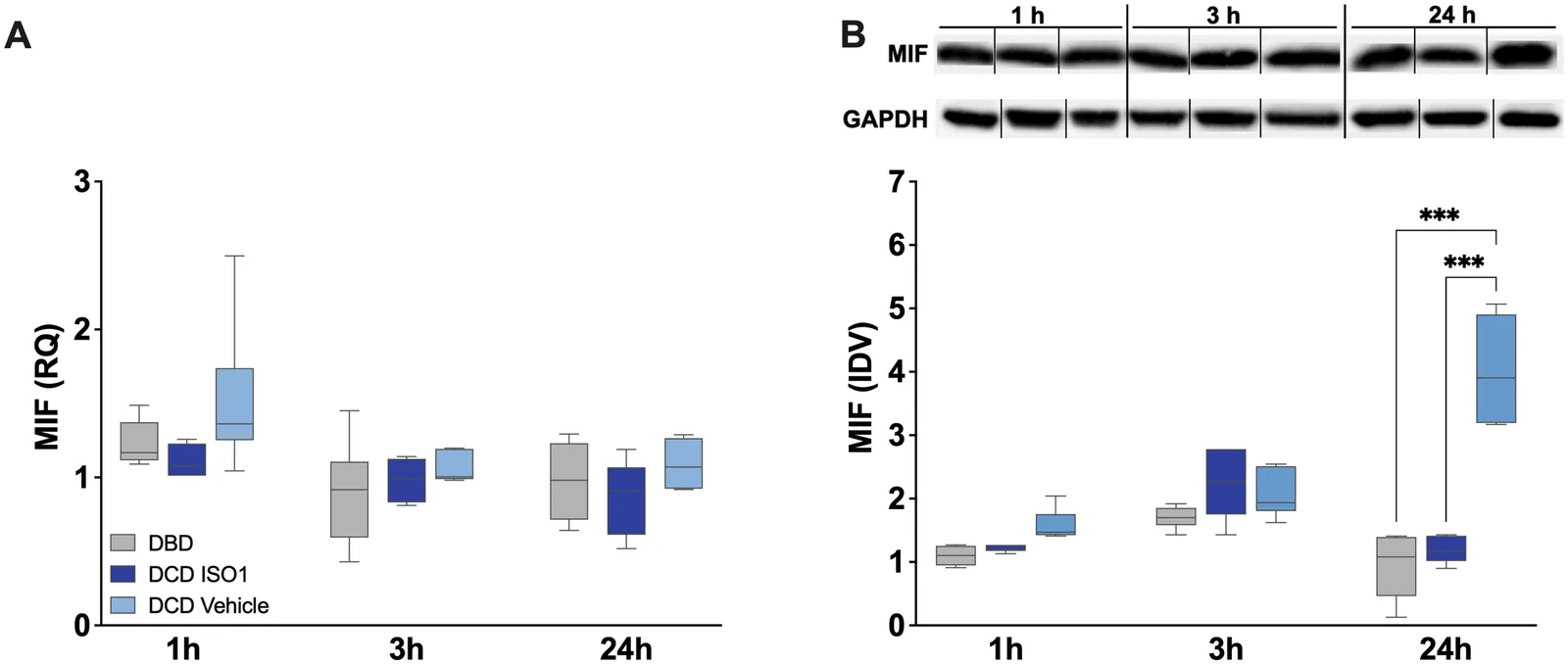
MIF mRNA (A) and tissue protein expression(B). *p < 0.05, **p < 0.01, ***p < 0.001; values are expressed as mean ± standard error of the mean; n = 7/group/time point. Abbreviations used: MIF-Macrophage migration inhibitory factor; RQ-Relative quantification; IDV-Integrated density value; The vertical lines in the Western blot images denote that the blots have been grouped to remove the triplicate lanes. Western blot analysis shown is representative of independent experiments. Complete raw data has been provided in the online supplement.

Serum MIF levels of the DCD Vehicle group have shown a strong increase in the early phase of reperfusion after 1 hours and a steep drop to markedly lower values after 3 and 24 hours, with a significant difference between ISO-1 and Vehicle groups at one hour (Serum MIF: DCD ISO-1_1hour_ vs DCD Vehicle_1hour_, 45 ± 5.3 vs 144 ± 27.5 ng/mL, p = 0.034, Fig 4).

Liver tissue MIF protein levels have interestingly shown a marked secondary increase after 24 hours of reperfusion in the vehicle group (MIF protein: DCD ISO-1_168hours_ vs DCD Vehicle_168hours,_ 1.2 ± 0.1 vs 4.0 ± 0.3 IDV, p = 0.0001, Fig 6 and S1 Images).

## Discussion

This study provides the first evidence that donor pre-treatment with the small-molecule MIF inhibitor ISO-1 confers significant protection against ischemia-reperfusion injury (IRI) in a rat model of donation after circulatory death (DCD) and arterialized orthotopic liver transplantation (OLT). Our findings demonstrate that ISO-1 administration prior to cardiac arrest substantially mitigates hepatocellular injury, improves graft macro- and microcirculation, reduces levels of the damage-associated molecular pattern HMGB-1 and serum MIF, and was associated with an altered time course of CD163-positive macrophage infiltration.

Sterile inflammation plays an important role in mediating IRI induced liver damage in DCD liver transplantation [20,21]. Although it has been demonstrated by our group and by others that early MIF activation can exhibit certain protective effects in organs such as the heart and the kidney [12–14], it was also shown that during liver IRI that MIF mostly plays a detrimental role by activating Kupffer-cells and promoting proinflammatory cytokine release [22,23]. However, the evidence from well-designed experimental models in the setting of OLT is missing (see Table 2).

**Table 2:** Studies in the context of MIF and liver transplantation.

Author	Species and strain	CIT & WIT	Sample size	Details	Time points	Short summary
*Uluk, Czigany et al.* *(present study)*	Rat, Lewis, inbred, male	3 h CIT 30 min WIT	154 rats, 77 OLT	Arterialized whole graft OLT with 30 min DCD and ISO-1 MIF inhibitor or Vehicle	1, 3, 24, 168 hours	“ISO-1 has led to the potent attenuation of IRI, improved microcirculation, and reduction of MIF serum levels.”
*Emontzpohl, Czigany et al. 2019 [22]*	Rat, Lewis, inbred, male	8 h CIT no WIT	144 rats, 72 OLT	Arterialized whole graft OLT with remote conditioning	1, 3, 24, 168 hours	“1, Remote ischemic conditioning led to a decrease of MIF on different levels, including liver tissue mRNA and tissue and serum protein. 2, MIF protein levels showed an association with the conventional surrogate markers of hepatocellular injury following OLT.”
*Liu et al. 2012 [34]*	Rat, Lewis, inbred, male	6 h CIT no WIT	12 rats, 6 OLT	Non-arterialized whole graft OLT	24 hours	The study suggests a role of MIF in mediating the inflammatory response to IRI.

Literature search (PubMed; search date: 08^th^ of December, 2025; search terms: MIF OR Macrophage Migration Inhibitory Factor AND liver transplantation) resulted in only two relevant studies. According to the best of our knowledge, the present study is the only experimental work using ISO-1 MIF inhibitor in the context of OLT. Abbreviations used: CIT-Cold ischemia time, WIT-Warm ischemia time; OLT-Orthotopic liver teansplantation; MIF-Macrophage Migration Inhibitory Factor; mRNA-messenger Ribonucleic acid.

To establish the ideal conditions to investigate the effects of ISO-1, a model of a moderate and reversible IRI is ideal, in which the most prominent therapeutic effects can be expected. On the one hand, in the setting of very mild injury, no therapeutic intervention will lead to a substantial and statistically significant outcomes, while on the other hand if IRI is extreme and lethal, any intervention will remain futile [24,25]. Therefore, in Experiment I the models of 30 and 60 minutes of cardiac arrest were compared with a “DBD” model without significant warm ischemia in the donor. Grafts were transplanted in a brief 1-hour reperfusion period. In this experiment we could observe a massive tissue injury in the grafts which were retrieved after 60 minutes of cardiac arrest and warm ischemia with extreme transaminases and LDH increase. Meanwhile, the DCD 30 group has shown a moderate increase in serum transaminases and LDH which was tendentially higher compared to the DBD group. In line with the marked hepatocellular damage, parameters of graft circulation have shown poor results after 60 minutes of warm ischemia. Based on these experiments and on the findings of other groups [25], the model of 30 minutes cardiac arrest was selected as model for Experiment II.

The small molecule ISO-1 ((S,R)-3-(4-hydroxyphenyl)-4,5-dihydro-5-isoxazole acetic acid methyl ester) was developed as an inhibitor of the keto-enol tautomerase activity of MIF [26] and has been used in numerous studies following intraperitoneal application in rats [27]. To achieve the most potent effects, we administered ISO-1 before the ischemic insult in the donor animals. Catalytic and functional inhibition are not identical: the tautomerase activity appears largely vestigial, whereas the pro-inflammatory actions of MIF are initiated through CD74/CD44 and CXCR2/CXCR4 [28,29], and compounds dissociating catalytic from functional inhibition exist [30]. ISO-1 reduces cytokine release from wild-type but not from MIF-deficient macrophages [26]. Its protection should therefore be read as an overall attenuation of MIF signaling rather than as blockade of an enzymatic activity as such.

Impairment of graft circulation both on the level of macro- and microcirculation is an important element in IRI in the liver transplantation setting [16,22]. We used the O2C device in multiple studies to investigate graft circulation in organ transplantation [16,22]. In Experiment II, animals of the vehicle group have shown a worsening of microcirculatory flow and velocity with a most prominent difference compared to ISO-1 and DBD groups after 24 hours of reperfusion. In contrast to graft microcirculation, PVF has shown more obvious differences in the early reperfusion with the lowest values in the vehicle group. There are multiple mechanisms contributing to the disturbances of allograft macro- and microcirculation following IRI, including, inflammation, endothelial cell swelling, neutrophil stasis, sludge and microthrombosis [31]. Even though, there is no direct evidence which could confirm our findings and would also show the direct effect of ISO-1 or MIF inhibition on tissue microcirculation, based on previous studies it is a plausible assumption that MIF, due to its early and rapid proinflammatory effects, contributes to microcirculatory dysfunction and failure [31,32]. A comparable direction of effect was reported by Gregory et al. in a non-hepatic model, in which MIF-deficient mice showed fewer leukocyte-endothelial interactions on intravital microscopy; this remains an indication from a different setting rather than a confirmation of our own findings [22,32]. No mediator of microcirculatory failure was assessed directly: leukocyte-endothelial interactions, endothelial activation markers, sinusoidal integrity and microthrombus formation were not measured. We therefore regard the microcirculatory improvement as a robust observation, but not as a mechanistically resolved effect.

Ischemia-reperfusion injury serves as trigger in inducing MIF and other pro-inflammatory mediators which then lead to hepatocellular injury [6]. In contrast to organs such as the heart and kidney where MIF plays an either protective or biphasic role [12,14,33], there is convincing evidence suggesting its predominantly detrimental effects in liver IRI and OLT [6,8]. To investigate whether ISO-1 can reduce IRI, we have measured serum transaminases and LDH at various time points. While 30 minutes cardiac arrest and liver transplantation resulted in a prominent increase of AST, ALT and LDH in the DCD Vehicle group after 1 hour of reperfusion, this was potently reduced by ISO-1. This is in line with previous experimental and clinical findings, showing that MIF activations correlates with liver injury in IRI [8,22,34].

MIF appears to act in an organ- and context-dependent manner. It limits infarct size in the heart through CD74-dependent AMPK activation [35] and acts biphasically in the kidney [29], whereas in the liver the evidence indicates a predominantly detrimental role [22,23,30]. The liver combines large pre-formed hepatocyte MIF pools with the largest resident macrophage population of the body, behaving differently compared to other organs.

Especially in the setting of DCD liver transplantation, prolonged warm ischemia leads to the release of high-mobility group box-1 (HMGB-1) from damaged cells, acting as a potent danger-associated molecular pattern [36]. The release of HMGB-1 does not just serve as a biomarker for cellular injury, this important so-called alarmin/DAMP plays an important central role in IRI and amplifies sterile inflammation by activating Toll-like receptors and Receptor for Advanced Glycation End products-RAGE on Kupffer cells, endothelial cells, and infiltrating leukocytes [36]. This HMGB-1/RAGE signaling axis drives cytokine production, microvascular dysfunction, and oxidative injury, aggravating graft dysfunction [37].

The reduction of circulating HMGB-1 under ISO-1 admits two non-exclusive explanations. HMGB-1 is released passively from necrotic hepatocytes, and the lower transaminase, LDH and Suzuki values indicate a smaller necrotic cell mass, so that an injury-proportional fall would be expected. Alternatively, MIF may act upstream: in non-hepatic models MIF release precedes and is required for HMGB-1 release through CD74 and CXCR4 [38], and MIF-induced phosphorylation promotes HMGB-1 secretion via TLR4/NF-kappaB [39]. The concordant early peaks of both mediators fit such a loop, but our data establish an association without identifying its direction.

As Kupffer cells and infiltrating macrophages are among the key responders to DAMPs such as HMGB-1, and their phenotype influences whether injury is aggravated or repair is facilitated [40], we assessed CD163 expression by immunohistochemistry. CD163, a haemoglobin-haptoglobin scavenger receptor, is preferentially expressed on macrophages of the alternatively activated, so-called M2 phenotype [41]. CD163-positive cells were more numerous in the ISO-1 group at 24 hours, whereas after one week only the vehicle group showed persistently elevated numbers. This is compatible with an earlier onset and earlier resolution of restorative signaling under ISO-1, but requires caution. CD163 is a single marker: the M1/M2 nomenclature derives largely from in vitro polarization and does not describe the mixed activation states of injured tissue in vivo [42], and CD163-positive macrophages are not uniformly reparative, CD163 having been linked to severity and adverse outcome in liver disease [43]. A broader marker panel remains a priority for future studies.

Baron-Stefaniak et al. have measured MIF protein levels in the effluent and serum of 38 human liver grafts [8]. They proposed that MIF protein correlates with hepatocellular injury after liver transplantation. Liu et al. have shown an increased in MIF liver mRNA and serum protein levels compared to non-ischemic controls in a rat model of OLT [34]. In the present study we have observed a similar pattern as MIF serum levels have correlated with serum transaminases and HMGB-1 levels with a prominent peak in the vehicle group after 1 hours of reperfusion. The administration of ISO-1 obviously led to markedly reduced serum MIF levels. On the liver tissue level, we registered a tendance towards higher mRNA levels and a secondary peak of MIF protein after 24 hours of reperfusion. The dissociation between rapidly rising circulating protein and unchanged hepatic transcript is consistent with the secretion biology of MIF: it is constitutively synthesized, stored pre-formed in cytoplasmic pools, and released within minutes of a stress stimulus through a non-conventionalmroute [44]. An early post-reperfusion surge is therefore transcription-independent and would not be matched by a rise in hepatic mRNA. Two caveats apply. With sampling at 1, 3 and 24 hours, an early transient transcriptional response or a delayed compensatory upregulation may have escaped detection, the secondary rise in tissue MIF at 24 hours fitting such replenishment. Downstream signaling events of MIF inhibition, in particular the AKT, ERK1/2 and STAT3 cascades, were not assessed in this pilot study and are being addressed in a dedicated follow-up project.

The findings of our study have to be interpreted in the light of certain limitations. This study is the first report on the use ISO-1 MIF inhibitor in OLT. Therefore, in this pilot setting we were not able to carry out a much deeper exploration of the subcellular mechanisms behind the observed protective effects of ISO-1, thus our findings remain predominantly descriptive. The number of investigated time points and group sizes also represent a further limitation for the robustness of our observations. Due to ethical and pragmatic considerations, MIF inhibition during ex vivo machine perfusion, instead using it as donor therapy would carry a superior clinical potential which has not been explored here. A further limitation is the absence of loss-of-function validation. Although ISO-1 is inactive in MIF-deficient cells [26], off-target contributions cannot be formally excluded here.

However, even with these limitations the present study shows the potent anti-inflammatory effects of MIF inhibition in a DCD liver transplantation setting for the first time and might carry a strong translational potential. ISO-1 pre-treatment attenuated hepatocellular injury and was associated with improved graft microcirculation and reduced DAMP release, supporting further mechanistic and translational evaluation in DCD liver transplantation. More detailed exploration of the mechanistic steps behind these observations are warranted in future studies. These would require a more complex layered experimental design and human translational aspects.

## Supporting information

S1 Images Raw blot images. (PDF)

S2 Table Score Sheet. (PDF)

## Abbreviations

ALT: alanine aminotransferase
ARRIVE: Animal Research: Reporting of In Vivo Experiments
AST: aspartate aminotransferase
ATP: adenosine triphosphate
DBD: donation after brain death
DCD: donation after circulatory death
DMSO: dimethylsulfoxide
ELISA: enzyme-linked immunosorbent assay
H&E: hematoxylin and eosin
HTK: histidine-tryptophan-α-ketoglutarate
IRI: ischemia reperfusion injury
ISO-1: (S, R)-3-(4-hydroxyphenil)-4,5-dihydro-5-isoxazole acetic acid methyl ester
LDH: lactate dehydrogenase
MIF: macrophage migration inhibitory factor
mRNA: messenger ribonucleic-acid
OLT: orthoptic liver transplantation
PVF: portal venous flow
RT-PCR: Reverse-Transcription Polymerase Chain Reaction
WB: western blotting
WIT: warm ischemia time
