# Supplementary material for: MIF inhibitor ISO-1 attenuates ischemia-reperfusion injury in a rat model of donation after circulatory death liver transplantation
Source: PLoS One. 2026 Oct 26;21(10):e0361214. doi: 10.1371/journal.pone.0361214 (PMC13704867; doi:10.1371/journal.pone.0361214)

Gel1

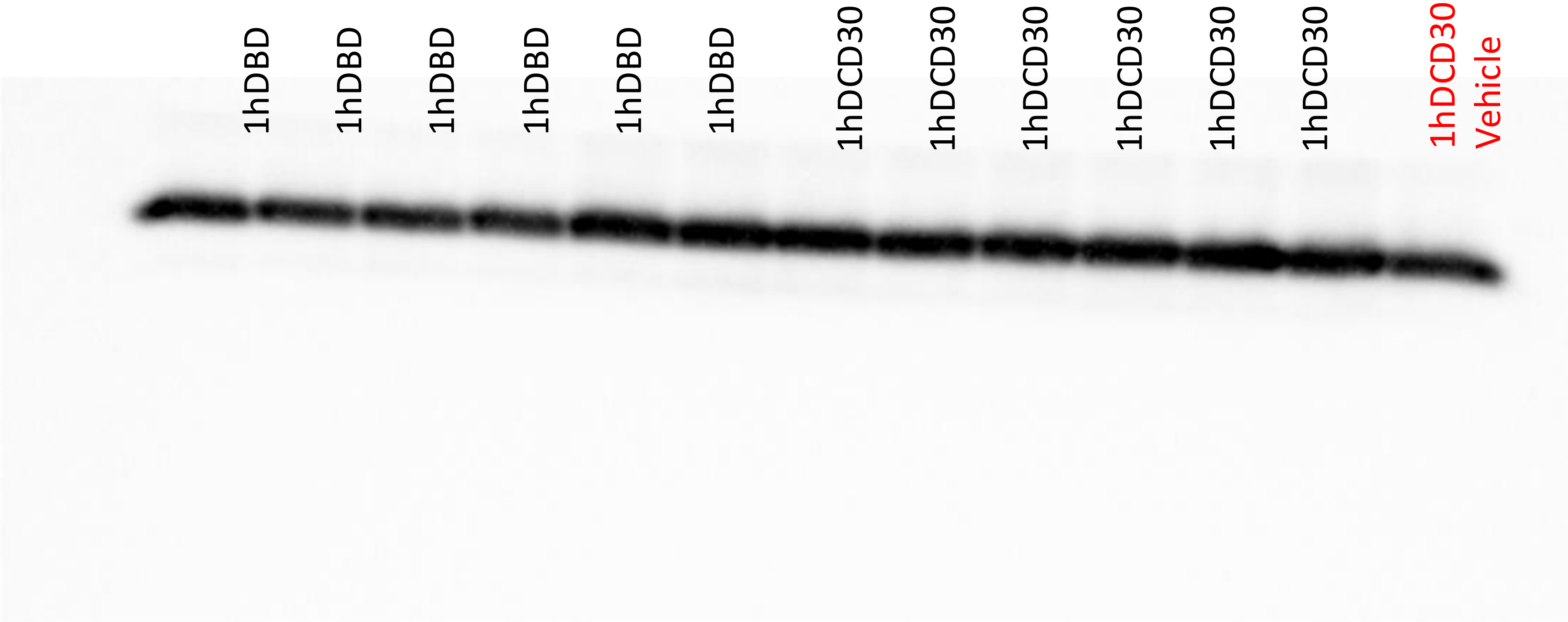

1hDBD
1hDBD
1hDBD
1hDBD
1hDBD
1hDBD
1hDCD30
1hDCD30
1hDCD30
1hDCD30
1hDCD30
1hDCD30
1hDCD30
Vehicle

Gel2

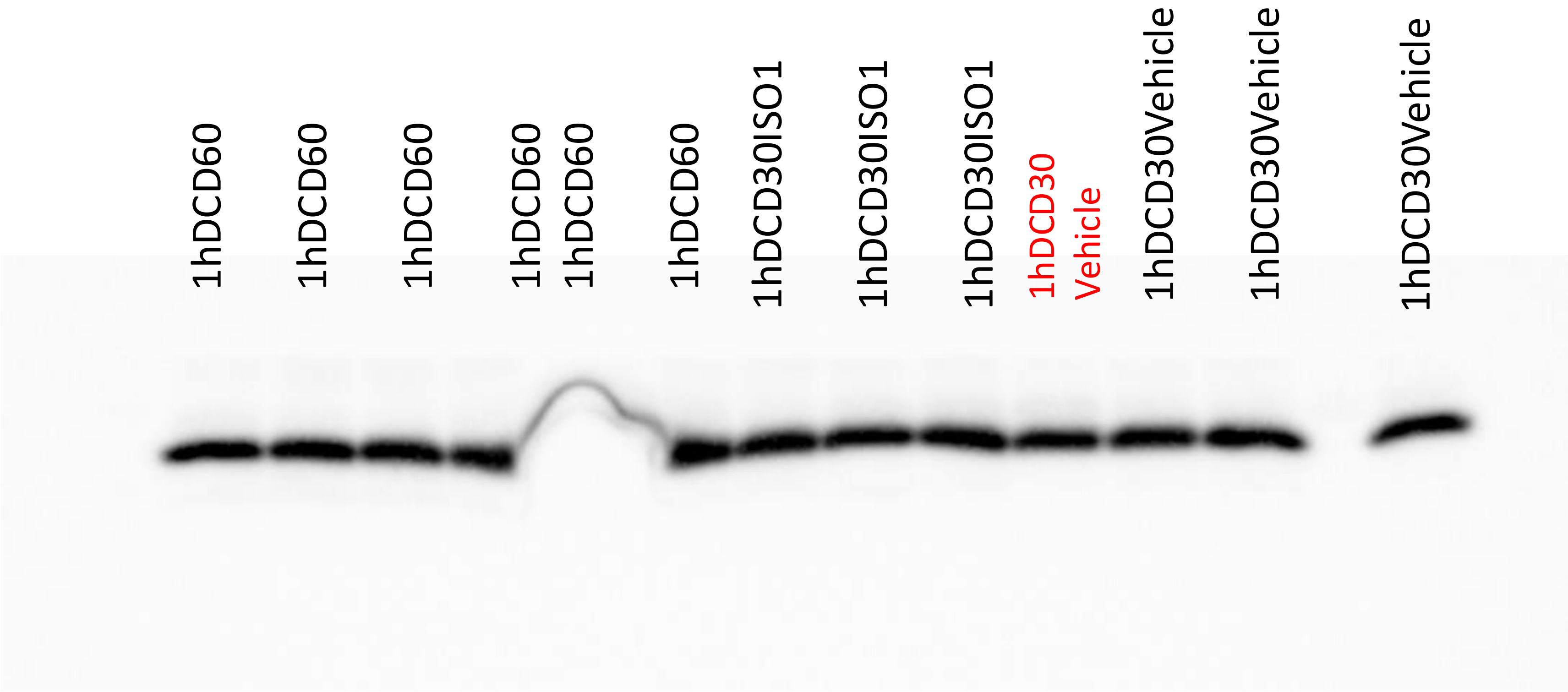
1hDCD60
1hDCD60
1hDCD60
1hDCD60
1hDCD60
1hDCD60
1hDCD30ISO1
1hDCD30ISO1
1hDCD30ISO1
1hDCD30 Vehicle
1hDCD30Vehicle
1hDCD30Vehicle
1hDCD30Vehicle

Gel3

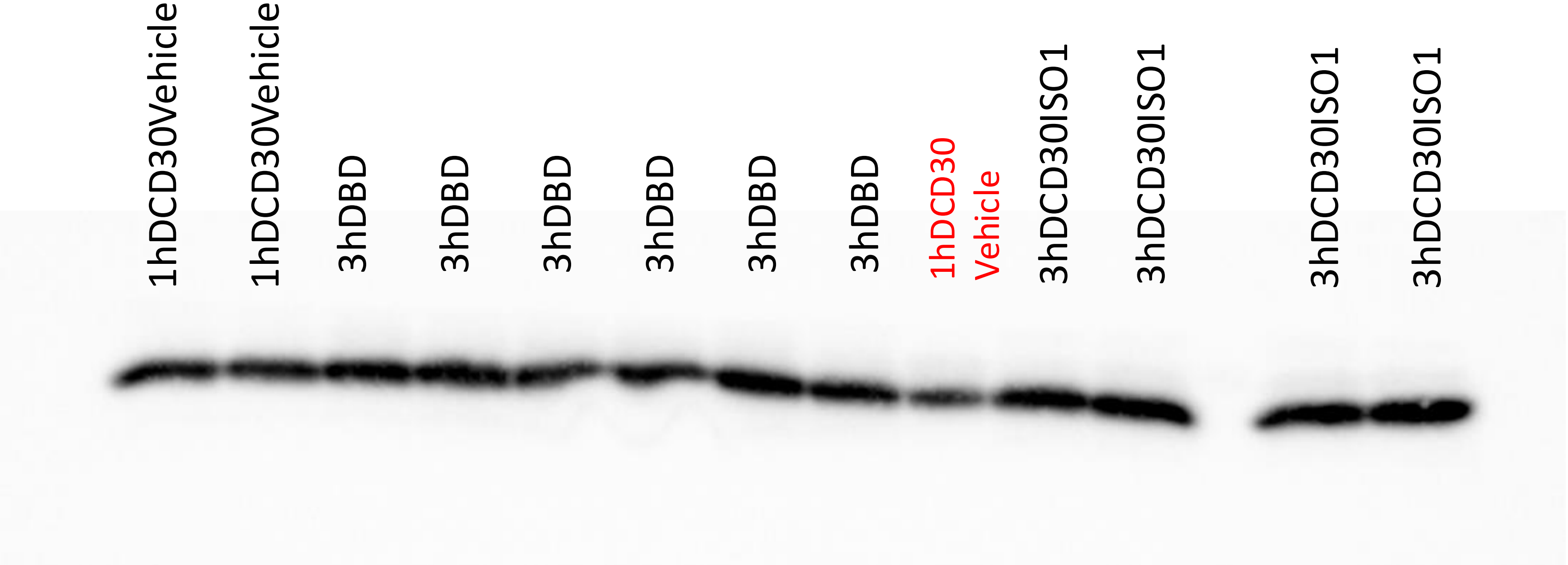

1hDCD30Vehicle
1hDCD30Vehicle
3hDBD
3hDBD
3hDBD
3hDBD
3hDBD
3hDBD
1hDCD30 Vehicle
3hDCD30ISO1
3hDCD30ISO1
3hDCD30ISO1
3hDCD30ISO1

Gel4

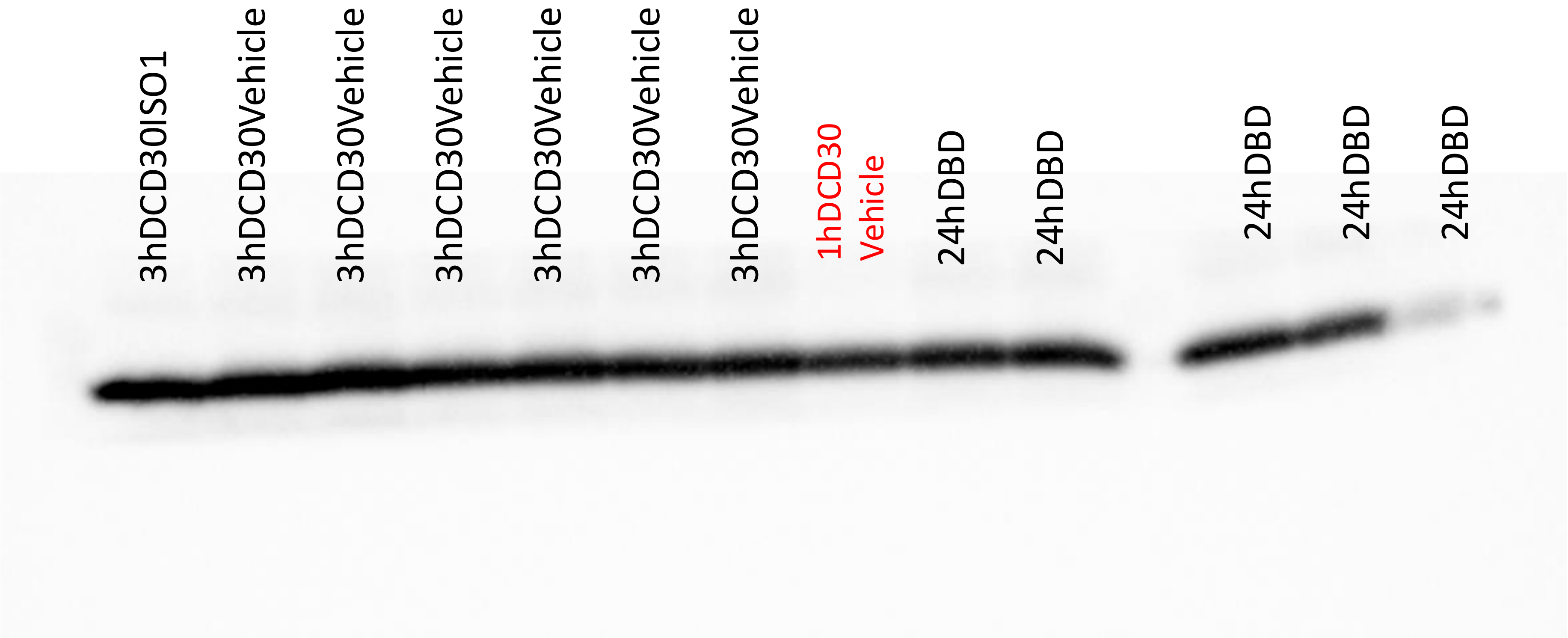
3hDCD30ISO1
3hDCD30Vehicle
3hDCD30Vehicle
3hDCD30Vehicle
3hDCD30Vehicle
3hDCD30Vehicle
3hDCD30Vehicle
1hDCD30 Vehicle
24hDBD
24hDBD
24hDBD
24hDBD
24hDBD

Gel5

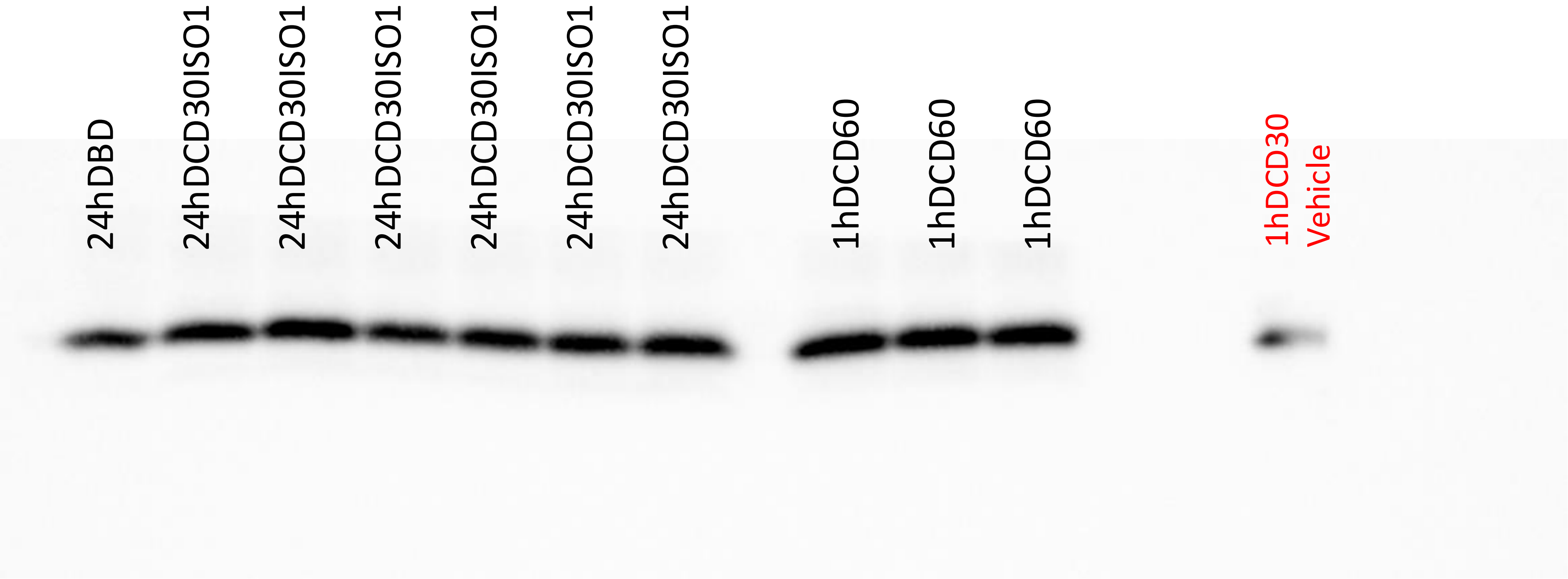
24hDBD
24hDCD30ISO1
24hDCD30ISO1
24hDCD30ISO1
24hDCD30ISO1
24hDCD30ISO1
24hDCD30ISO1
1hDCD60
1hDCD60
1hDCD60
1hDCD30
Vehicle

Gel6

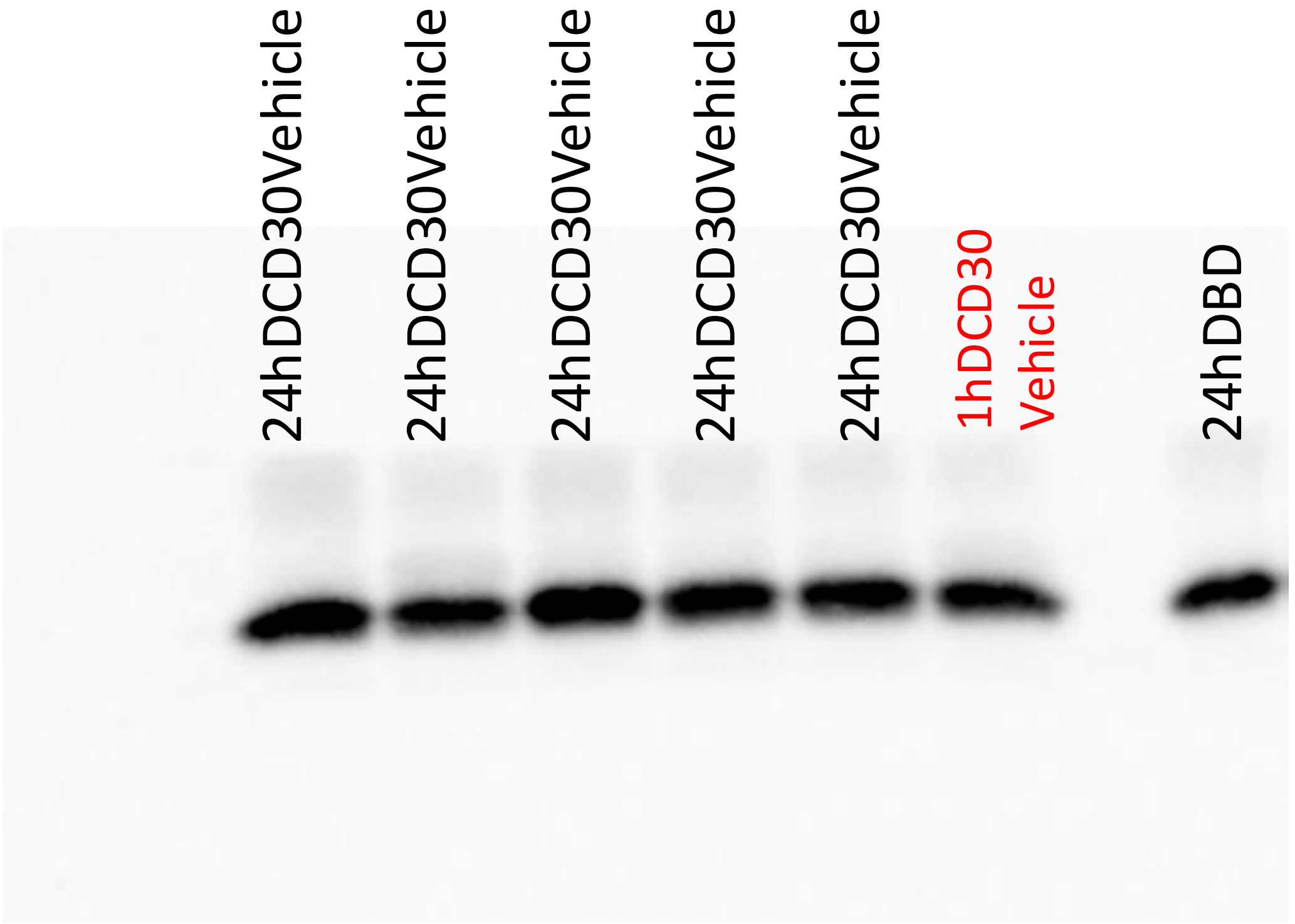
24hDCD30Vehicle
24hDCD30Vehicle
24hDCD30Vehicle
24hDCD30Vehicle
24hDCD30Vehicle
1hDCD30 Vehicle
24hDBD

Gel1

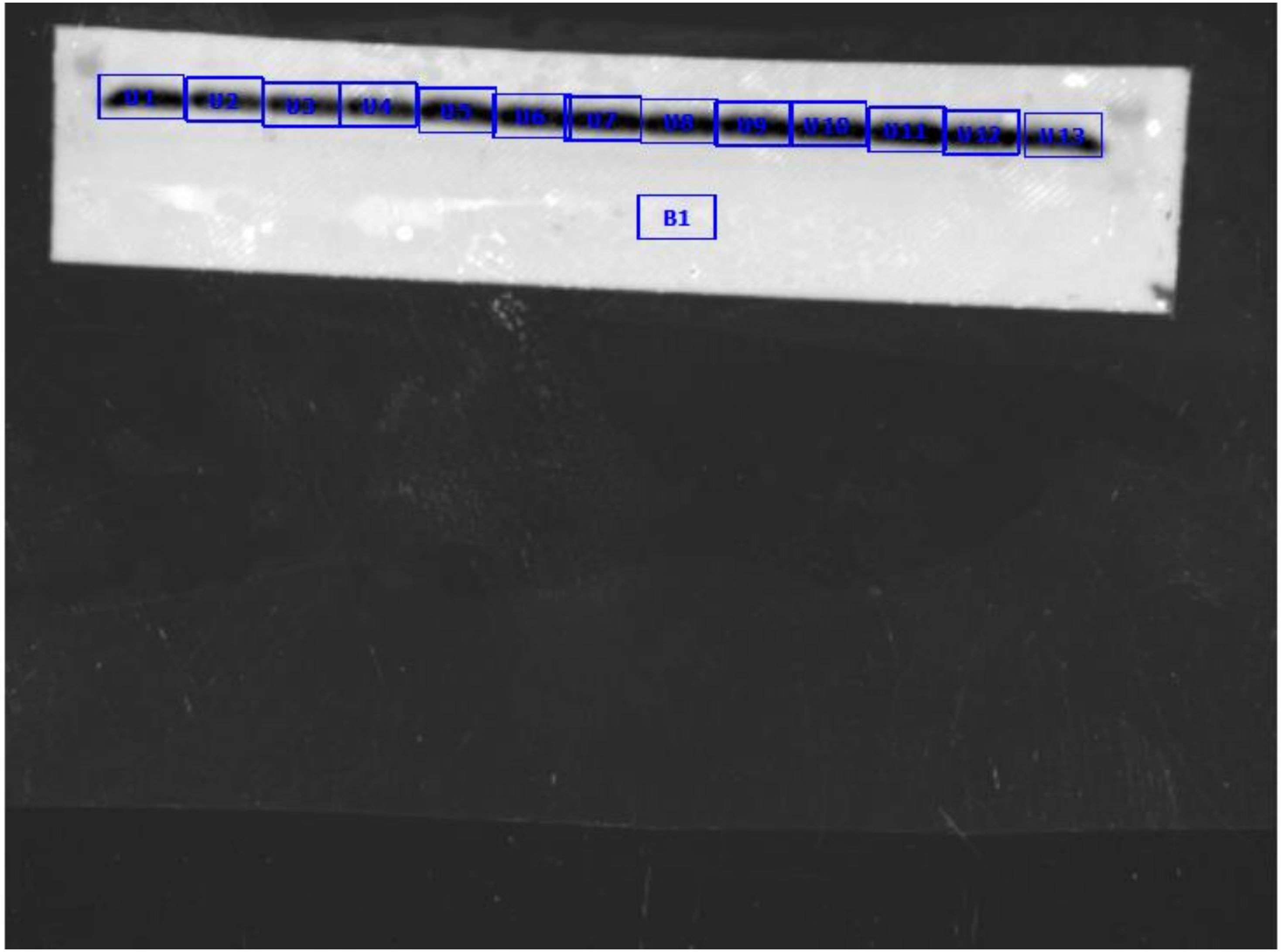

Gel2

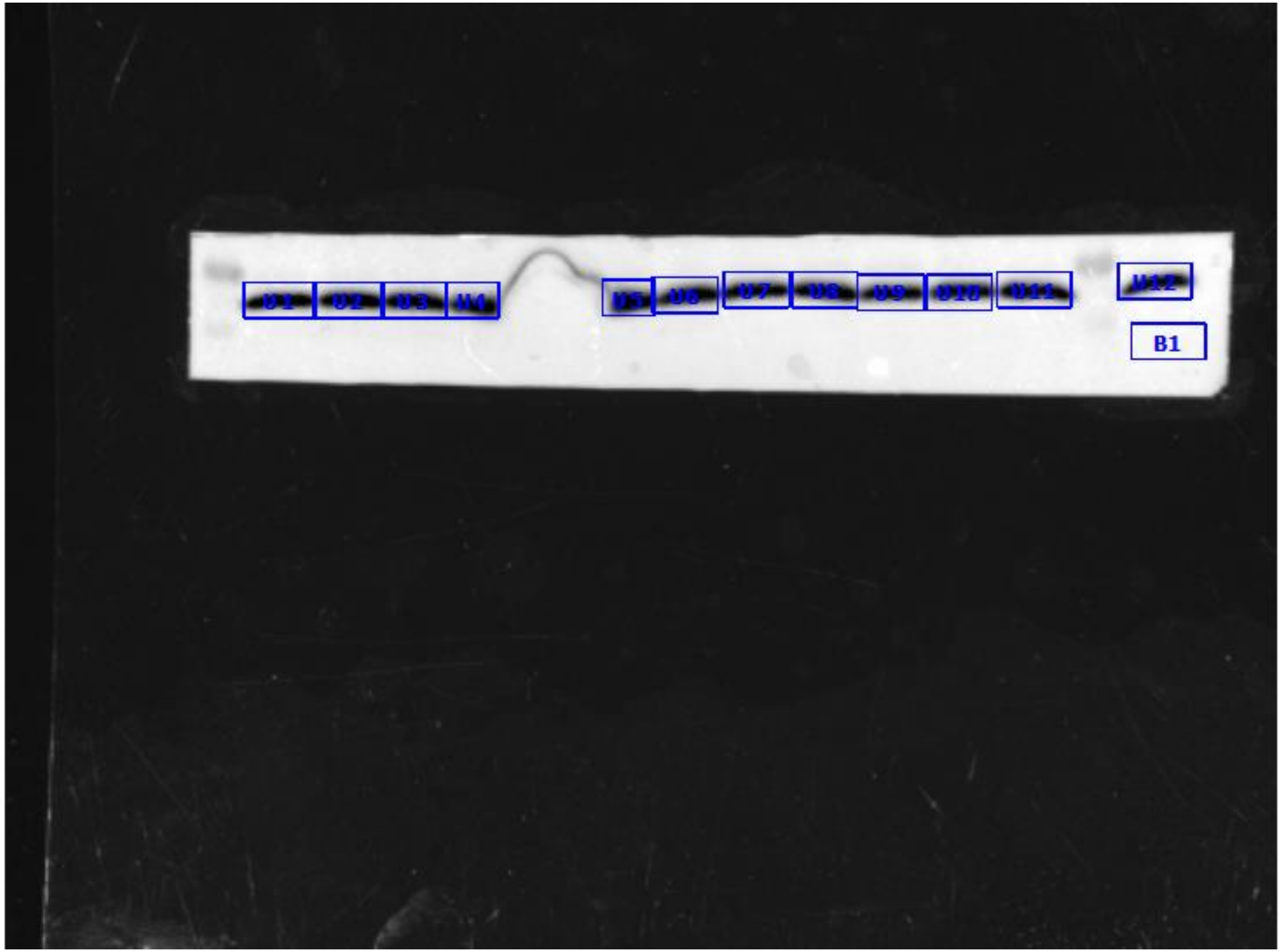

Gel3

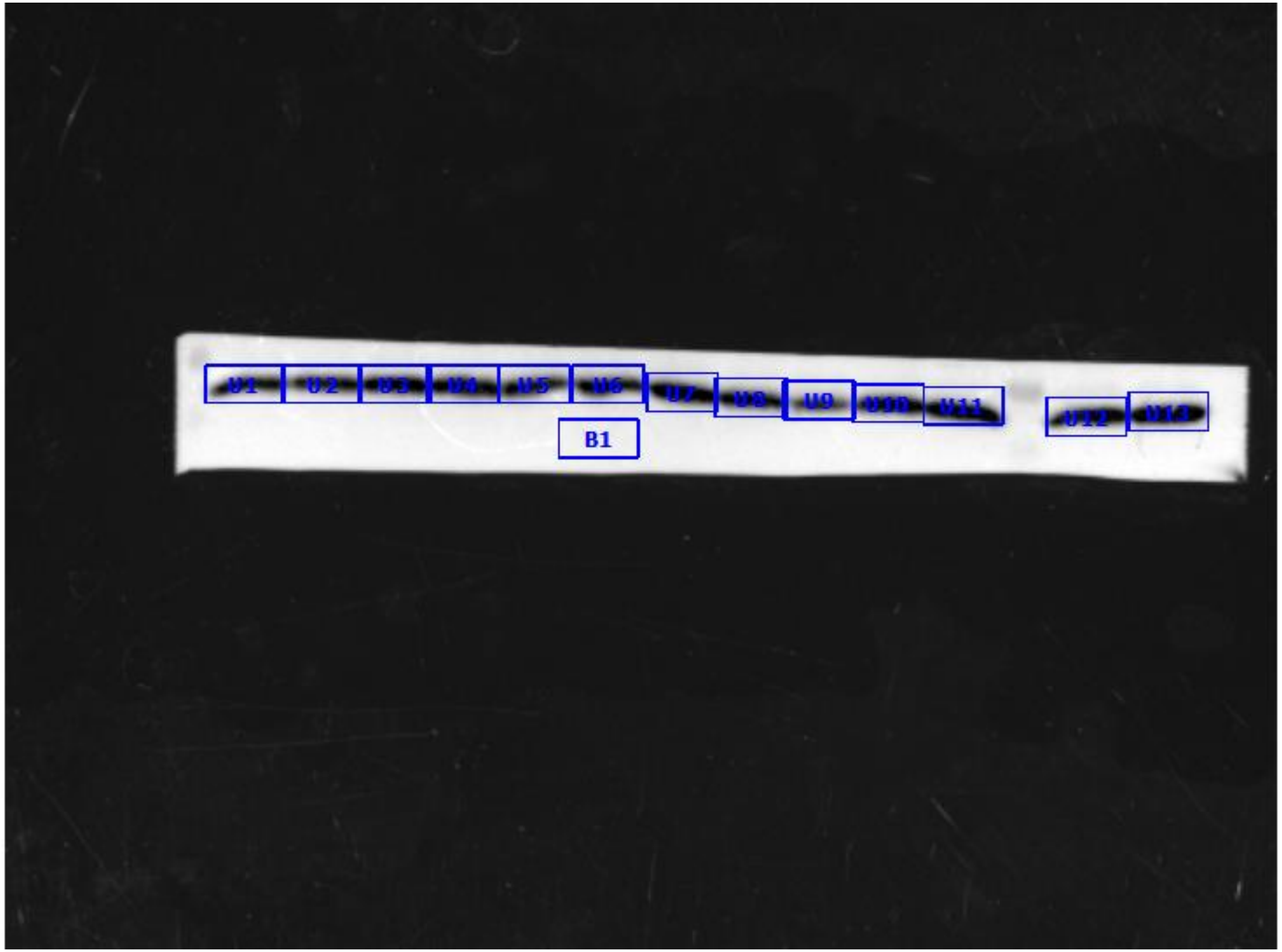

Gel4

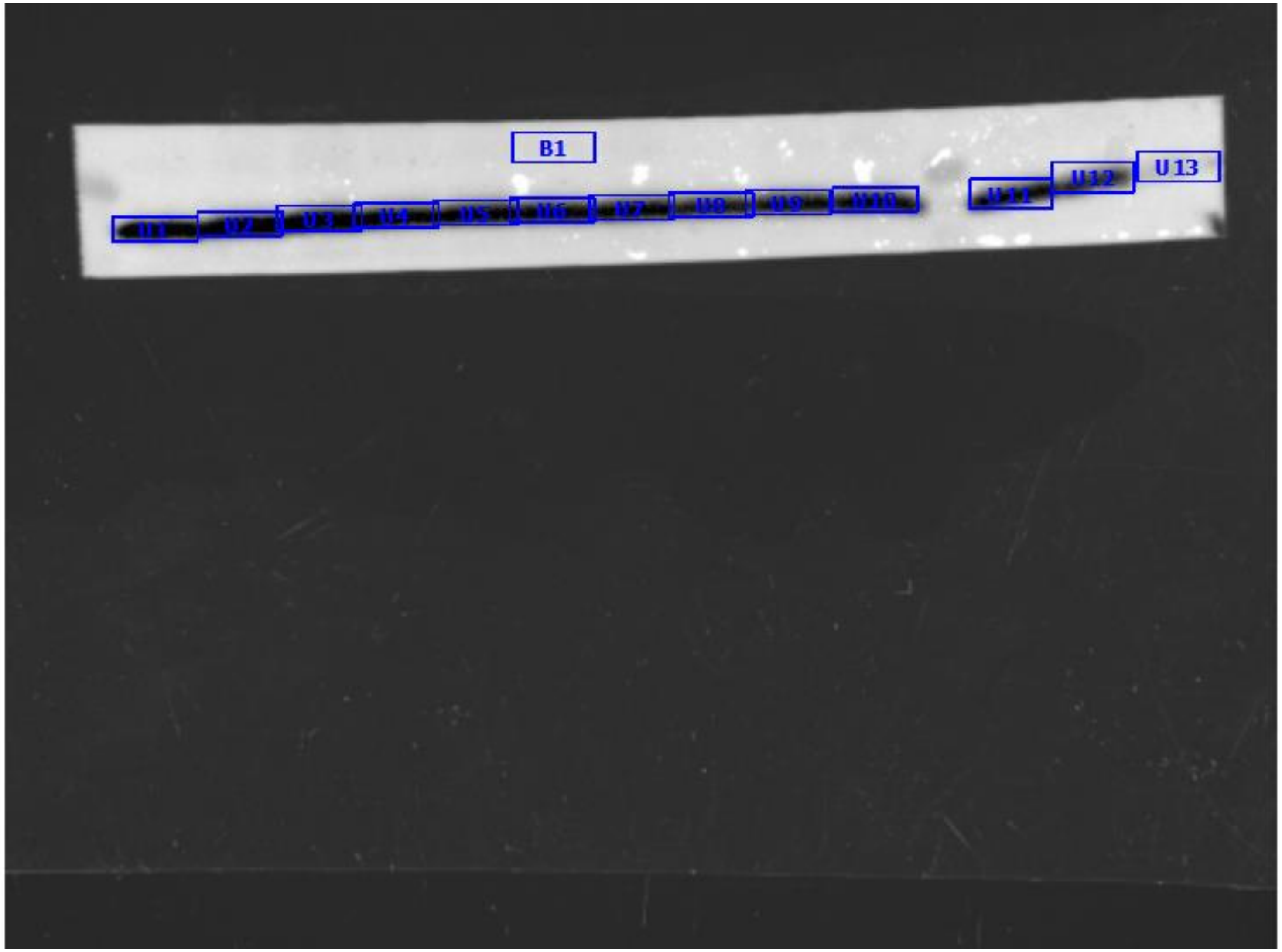

Gel5

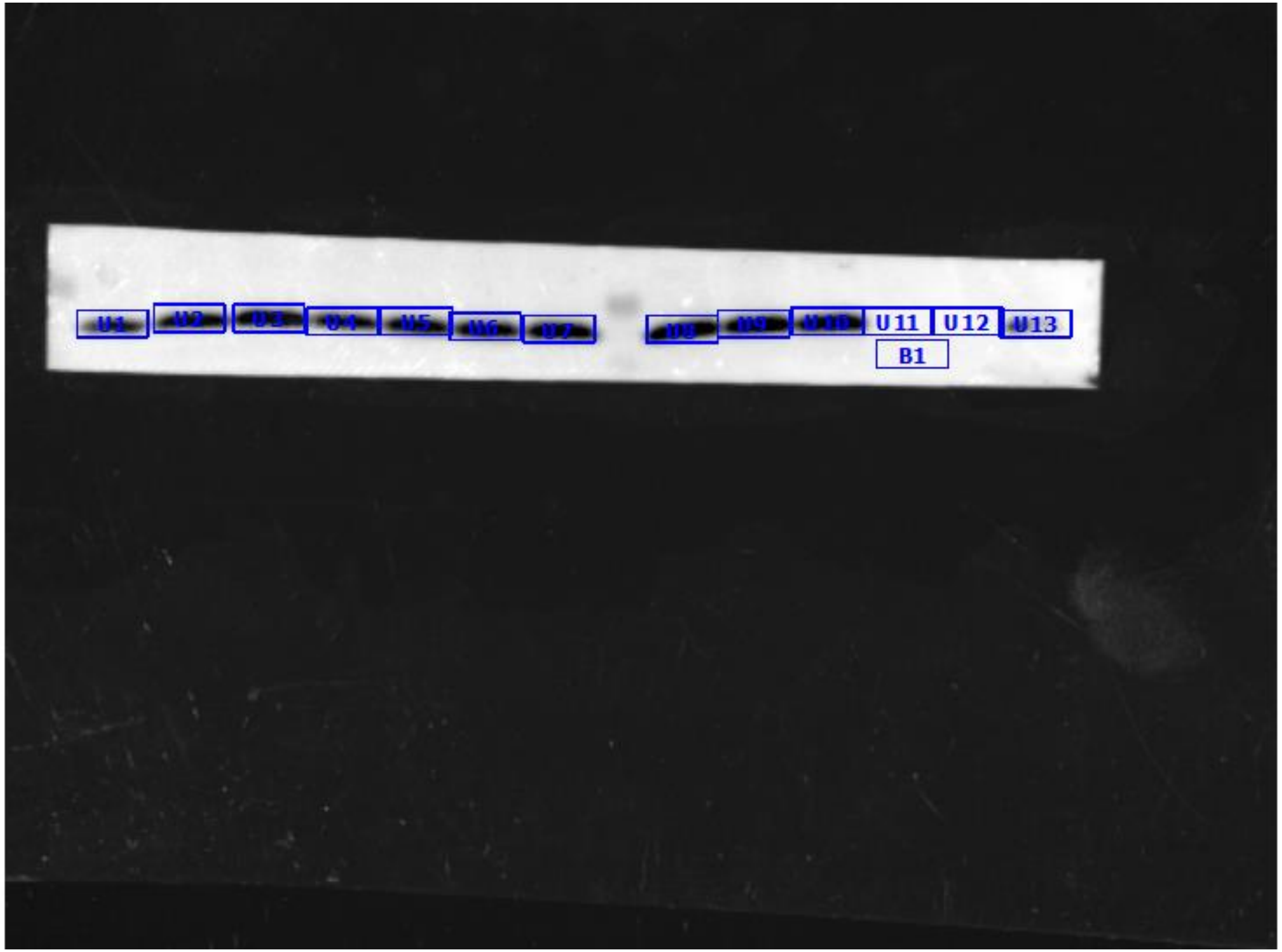

Gel6

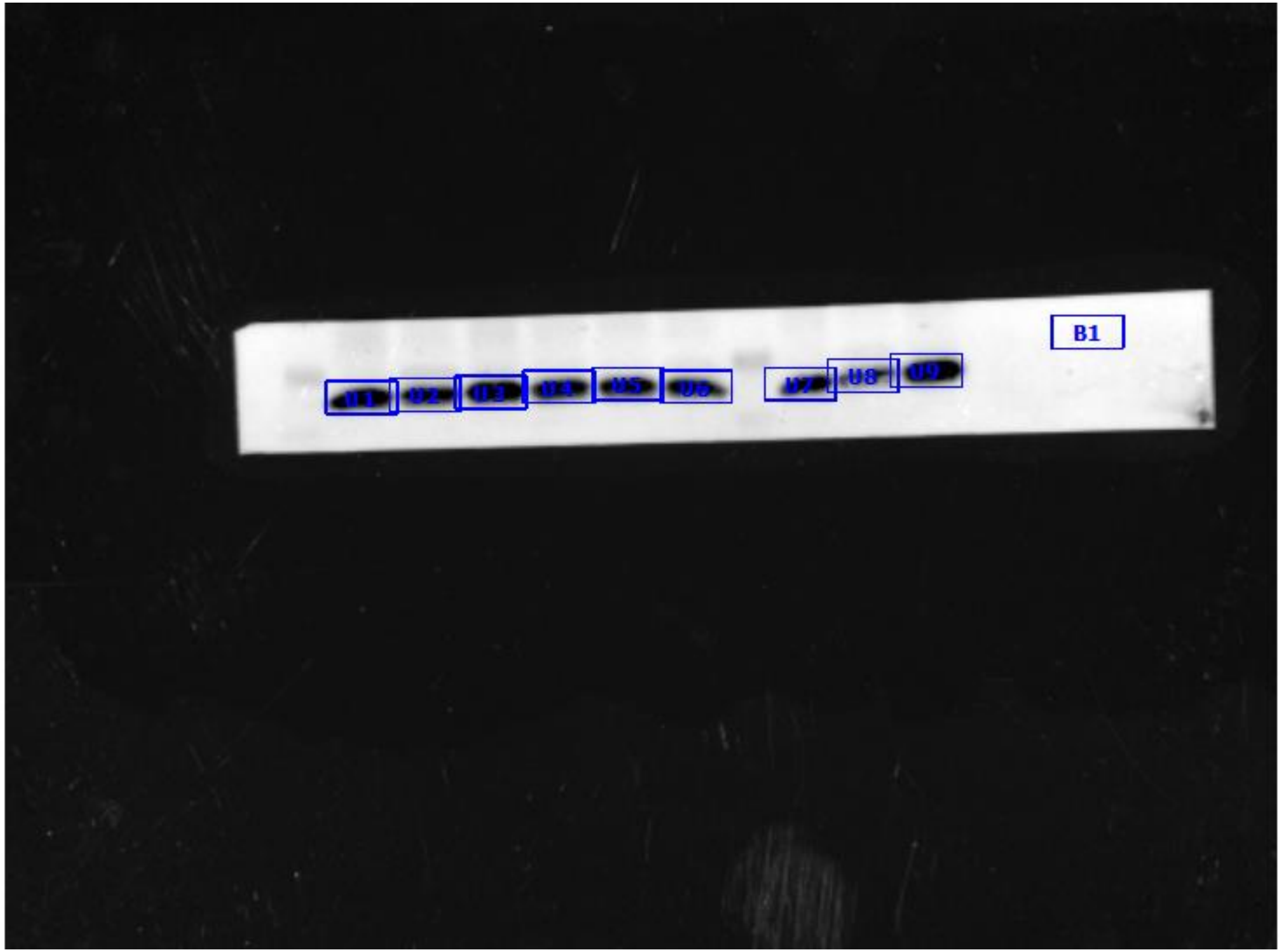

Supplement: Supplementary file 1 [file pone.0361214.s001.pdf]
