# Supplementary material for: MIF inhibitor ISO-1 attenuates ischemia-reperfusion injury in a rat model of donation after circulatory death liver transplantation
Source: PLoS One. 2026 Oct 26;21(10):e0361214. doi: 10.1371/journal.pone.0361214 (PMC13704867; doi:10.1371/journal.pone.0361214)
Supplement: Supplementary file 2 [file pone.0361214.s002.pdf]

Appendix Table 3. Score sheet with specific humane-endpoint (termination) criteria

Source: Kanzler, …, Czigany, et al. *Laboratory Animals* 2016.

Observation	Score
**I Body weight**	
• Unaffected or increase	0
• Change < 5%	1
• Weight reduction 5–10%	5
• Weight reduction 11–19%	10
• Weight reduction ≥ 20%	20
**II General condition**	
• Coat smooth and shiny; body orifices clean; eyes clear and bright	0
• Coat defects (reduced or excessive grooming)	1
• Coat dull and unkempt, unclean body orifices, eyes cloudy; increased muscle tone, minimal wound-healing disturbance or small subcutaneous seroma	5
• Dirty coat, matted or moist body orifices, abnormal posture, cloudy eyes; high muscle tone, moderate wound-healing disturbance or larger subcutaneous seroma, clinically moderate ascites (signs of increased abdominal girth due to fluid accumulation)	10
• Cramps/spasms, paralysis (trunk musculature, extremities); abnormal respiratory sounds; or repeated suture dehiscence or pronounced wound-healing disturbance; jaundice as a sign of liver failure or cholangitis; clinically severe ascites (clearly increased abdominal girth)	20
**III Spontaneous behaviour**	
• Normal behaviour (sleeping, reaction to blowing and touch, curiosity, social contact)	0
• Minimal deviations from normal behaviour	1
• Unusual behaviour, restricted motor function or hyperkinesia	5
• Self-isolation, lethargy; pronounced hyperkinesia or behavioural stereotypies; coordination disorders	10
• Vocalisation of pain when handled; self-amputation (auto-aggression)	20
**IV Clinical findings**	
• Temperature (measured in conspicuous animals with an infrared thermometer), respiration normal, extremities warm, mucous membranes well perfused	0
• Minor deviations from the normal situation	1
• Slight variation in temperature 1–2 °C; or respiration increased (~30%), extremities clearly colder than normal, reduced mucous-membrane perfusion	5
• Pronounced variation in temperature >2 °C; or respiration markedly increased (~50%), extremities cold, poorly perfused mucous membrane or cyanosis	20
**Assessment and measures**	**Total score**
**Severity grade 0** (no distress)	0
**Severity grade 1** (minimal distress): continue to observe	1–9
**Severity grade 2** (moderate distress): initiate veterinary care if necessary (analgesia, wound care); consult the animal-welfare officer if necessary; with a pronounced overall picture, consider terminating the experiment (only as an individual, case-based decision together with the animal-welfare officer)	10–19
**Severity grade 3** (severe distress): consult the animal-welfare officer; terminate the experiment immediately, initiate veterinary care, euthanise the animal	≥20
